# Effect of fat-reformulated dairy food consumption on postprandial flow-mediated dilatation and cardiometabolic risk biomarkers compared with conventional dairy: a randomized controlled trial

**DOI:** 10.1093/ajcn/nqab428

**Published:** 2022-01-10

**Authors:** Oonagh Markey, Dafni Vasilopoulou, Kirsty E Kliem, Colette C Fagan, Alistair S Grandison, Rachel Sutton, David J Humphries, Susan Todd, Kim G Jackson, David I Givens, Julie A Lovegrove

**Affiliations:** Hugh Sinclair Unit of Human Nutrition and Institute for Cardiovascular and Metabolic Research, University of Reading, Reading, United Kingdom; Hugh Sinclair Unit of Human Nutrition and Institute for Cardiovascular and Metabolic Research, University of Reading, Reading, United Kingdom; Animal, Dairy, and Food Chain Sciences, University of Reading, Reading, United Kingdom; Institute for Food, Nutrition, and Health, University of Reading, Reading, United Kingdom; Hugh Sinclair Unit of Human Nutrition and Institute for Cardiovascular and Metabolic Research, University of Reading, Reading, United Kingdom; Institute for Food, Nutrition, and Health, University of Reading, Reading, United Kingdom; Hugh Sinclair Unit of Human Nutrition and Institute for Cardiovascular and Metabolic Research, University of Reading, Reading, United Kingdom; Hugh Sinclair Unit of Human Nutrition and Institute for Cardiovascular and Metabolic Research, University of Reading, Reading, United Kingdom; Hugh Sinclair Unit of Human Nutrition and Institute for Cardiovascular and Metabolic Research, University of Reading, Reading, United Kingdom; Institute for Food, Nutrition, and Health, University of Reading, Reading, United Kingdom; Department of Mathematics and Statistics, University of Reading, Reading, United Kingdom; Hugh Sinclair Unit of Human Nutrition and Institute for Cardiovascular and Metabolic Research, University of Reading, Reading, United Kingdom; Institute for Food, Nutrition, and Health, University of Reading, Reading, United Kingdom; Institute for Food, Nutrition, and Health, University of Reading, Reading, United Kingdom; Hugh Sinclair Unit of Human Nutrition and Institute for Cardiovascular and Metabolic Research, University of Reading, Reading, United Kingdom; Institute for Food, Nutrition, and Health, University of Reading, Reading, United Kingdom

**Keywords:** apolipoprotein B, cardiovascular disease risk, dairy fat, food chain approach, monounsaturated fatty acids, postprandial lipemia, saturated fatty acids, sequential test meal protocol, vascular function, *trans* fatty acids

## Abstract

**Background:**

Longer-term consumption of SFA-reduced, MUFA-enriched dairy products has been reported to improve fasting flow-mediated dilatation (FMD). Yet, their impact on endothelial function in the postprandial state warrants investigation.

**Objectives:**

The aim was to compare the impact of a fatty acid (FA) modified with a conventional (control) dairy diet on the postprandial %FMD (primary outcome) and systemic cardiometabolic responses to representative meals, and retrospectively explore whether treatment effects differ by apolipoprotein E (*APOE*) or endothelial NO synthase (*eNOS*) Glu298Asp gene polymorphisms.

**Methods:**

In a crossover-design randomized controlled study, 52 adults with moderate cardiovascular disease risk consumed dairy products [38% of total energy intake (%TE) from fat: FA-modified (target: 16%TE SFAs; 14%TE MUFAs) or control (19%TE SFAs; 11%TE MUFAs)] for 12 wk, separated by an 8-wk washout. Blood sampling and FMD measurements (0–480 min) were performed pre- and postintervention after sequential mixed meals that were representative of the assigned dairy diets (0 min, ∼50 g fat; 330 min, ∼30 g fat).

**Results:**

Relative to preintervention (∆), the FA-modified dairy diet and meals (treatment) attenuated the increase in the incremental AUC (iAUC), but not AUC, for the %FMD response observed with the conventional treatment (–135 ± 69% vs. +199 ± 82% × min; *P* = 0.005). The ∆ iAUC, but not AUC, for the apoB response decreased after the FA-modified treatment yet increased after the conventional treatment (–4 ± 3 vs. +3 ± 3 mg/mL × min; *P* = 0.004). The ∆ iAUC decreased for plasma total SFAs (*P* = 0.003) and *trans* 18:1 (*P* < 0.0001) and increased for *cis*-MUFAs (*P* < 0.0001) following the conventional relative to the FA-modified treatment. No treatment × *APOE* or *eNOS* genotype interactions were evident for any outcome.

**Conclusions:**

This study provides novel insights into the longer-term effects of FA-modified dairy food consumption on postprandial cardiometabolic responses.

## Introduction

Dietary supplementation of the dairy cow diet with plant oils or oil seeds offers a strategy for partially replacing milk SFAs with unsaturated fatty acids (FAs), largely in the form of *cis*-MUFAs (1). This agriculturally based reformulation strategy has the potential to limit the entry of SFAs into the food chain, while preserving micronutrient and bioactive components of dairy foods (2–4). Our group previously demonstrated that 12-wk intake of FA-modified milk, cheese, and butter (∼41 g dairy fat/d) had a beneficial effect on fasting endothelium-dependent flow-mediated dilatation (FMD) and circulating nitrite concentrations among adults at moderate cardiovascular disease (CVD) risk, relative to conventional dairy (5).

The nonfasted state is also recognized as important in the context of cardiometabolic disease development and progression (6). Elevated postprandial triacylglycerol (TG) concentration (an independent CVD risk factor) after high-fat meal consumption can potentiate inflammatory events and induce transient endothelial dysfunction, which is reflective of a vascular phenotype prone to atherogenesis (7). We observed a tendency for a higher AUC for the %FMD response following acute exposure to sequential FA-modified dairy fat–rich meals, relative to conventional dairy (8). However, the FA composition of the background diet can also influence postprandial cardiometabolic responses and may be of greater importance to long-term health than isolated (acute) dietary FA exposures (9). Furthermore, genetic makeup has also been shown to impact postprandial cardiometabolic disease risk markers (10–12), with *APOE4* allele or endothelial NO synthase (*eNOS*) Glu298Asp (rs1799983) polymorphism found to be responsive to the replacement of meal SFAs with *cis*-MUFAs (13, 14). However, the impact of these genotypes on postprandial cardiometabolic risk outcomes in response to changes in habitual FA intake is unclear.

The purpose of this proof-of-concept acute-within-chronic study, therefore, was to evaluate, among adults at moderate CVD risk, the effect of a 12-wk, high-fat, high-dairy diet including SFA-reduced, MUFA-enriched dairy products on the postprandial cardiometabolic disease risk outcomes and the plasma total lipid FA profile to sequential high-fat mixed meals incorporating diet-specific dairy products. We hypothesized that longer-term consumption of FA-modified dairy products (milk, cheese, and butter) would improve the postprandial %FMD response (primary outcome) and other systemic cardiometabolic risk biomarker responses to sequential meals containing FA-modified dairy products, compared with a matched treatment containing dairy foods with an FA profile typical of conventional retail products (control). A secondary explorative objective of this study was to evaluate the impact of single nucleotide polymorphisms in the *APOE* and *eNOS* genes, determined retrospectively, on observed cardiometabolic responses.

## Methods

### Participants

The REplacement of SaturatEd fat in dairy on Total cholesterol (RESET) study (NCT02089035) was given a favorable ethical opinion for conduct by the Research Ethics Committee at the University of Reading (ref: 13/43) and conducted in accordance with the Declaration of Helsinki. All participants gave written informed consent before participation, including consent for the retrospective genotyping for *APOE* (rs7412 and rs429358) and *eNOS* (rs1799983).

Weight-stable men and women [aged 25–70 y; BMI (kg/m^2^): 19–32] with moderate risk of developing CVD were enrolled into the study, which was conducted at the Hugh Sinclair Unit of Human Nutrition, University of Reading (Berkshire, United Kingdom) between February 2014 and April 2016. Details of the eligibility criteria are presented elsewhere (5, 15). In brief, a modified Framingham CVD risk score was determined from fasted screening measures of serum total cholesterol (TC), HDL cholesterol, glucose, blood pressure, BMI, or waist circumference and family history of myocardial infarction (16, 17). To be deemed eligible for study entry, participants were required to have a risk score of ≥2 points, reflecting a moderate risk of CVD (≥50% above the population mean). Eligible participants were nonsmokers; not diagnosed with CVD or diabetes (and fasting glucose concentration <7 mmol/L); presented with mild/moderate hypercholesterolemia (fasting total cholesterol ≥5.2–8.0 mmol/L); not currently taking medication for hyperlipidemia, hypertension, hypercoagulation, or inflammatory disorders; not pregnant or lactating; not consuming excessive amounts of alcohol (<14 and 21 units/wk for women and men, respectively; 1 unit was defined as 10 mL or 8 g pure alcohol); not participating in excessive amounts of vigorous aerobic physical activity (<3 times × 20 min/wk); and who presented with normal biochemistry for liver and kidney function (15). Participants were asked to self-identify their ethnic group using the UK 2011 Census Categories (18).

### Study design and dietary intervention

**FIGURE 1:**
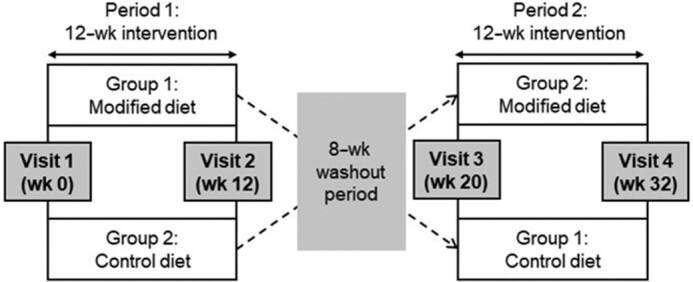
Overview of the RESET double-blind, crossover randomized controlled dietary trial. Participants were randomly assigned to group 1, where a postprandial visit (incorporating sequential meals rich in FA-modified dairy products) was completed before and after a 12-wk dietary intervention period with the same FA-modified dairy products (period 1), or group 2, where a postprandial visit [incorporating sequential meals rich in conventional (control) dairy products] was completed before and after a 12-wk dietary intervention period with the same control dairy products (period 1). Following an 8-wk washout period, participants crossed over to the alternate diet period and completed postprandial study visits before and after a 12-wk dietary intervention (period 2). FA, fatty acid; RESET, REplacement of SaturatEd fat in dairy on Total cholesterol.

The RESET intervention was a double-blind, crossover, randomized, controlled proof-of-concept study, where two 12-wk dietary intervention periods were separated by an 8-wk washout period. Fasting data from the RESET chronic intervention study have been published elsewhere (5). A minimization technique that stratified by age (25.0–40.0 or 40.1–55.1 or 55.2–70.0 y), sex (male or female), BMI (19.0–26.9 or 27.0–32.0 kg/m^2^), and fasting serum TC (≤5.9 or ≥6.0–8.0 mmol/L) was used by a single researcher (OM) to randomly allocate participants to their first treatment period, as previously described (5, 15). A 480-min postprandial study visit was performed at the beginning [preintervention: week 0, week 20; acute study results are presented in our earlier publication (8)] and end (postintervention: week 12, week 32) of each dietary period. This enabled us to examine the effect of longer-term dairy FA manipulation on postprandial endothelial function and systemic cardiometabolic responses to a sequential 2-meal dairy fat challenge (see **Figure 1**).

The details of our high-oleic sunflower (HOS) oil dairy cow supplementation approach, as well as the nutrient composition and FA profile of the modified and control dairy products, are presented elsewhere (15, 19, 20). For each 12-wk dietary intervention, participants were instructed to exchange their habitual dairy foods, cooking oil/spreads, and snacks with the SFA-reduced, MUFA-enriched (modified), or matched conventional (control) ultra-high temperature (UHT) milk, Cheddar cheese, and butter (∼41 g/d of dairy fat). The high-fat, high-dairy dietary exchange was isoenergetic [providing 38% of total energy (%TE) from total fat] but varied in FA composition (modified: 16%TE SFAs and 14%TE MUFAs; control: 19%TE SFAs and 11%TE MUFAs) (15). Participants were instructed to consume 340 g UHT milk/d, 45 g Cheddar cheese/d, and 25.1 g (FA-modified diet) or 21.5 g (control diet) butter/d. The products from both diets were similarly packaged and were masked by a single letter code (A: modified diet; B: control diet) by an investigator (CCF) not involved in the dietary intervention. We have shown that consumers generally accepted our FA-modified UHT milk, cheese, and butter in a blind taste-test study (20). The energy, macronutrient, and FA composition of the products has been published elsewhere (15, 20). Compliance was monitored using 4-d food diaries (pre- and postintervention) and daily consumption records, the results of which have been published (15). In addition, physical activity was assessed subjectively using the self-administered, last-7-d version of the International Physical Activity Questionnaire (IPAQ)–long form.

### Postprandial test meal protocol

The sequential 2-meal dairy fat challenge was conducted using dairy products identical to those assigned in the FA-modified and conventional (control) dietary intervention periods, as described previously (8). The energy and nutrient compositions of the sequential meals (breakfast and lunch) and test meal ingredients are outlined in **Table 1**. The breakfast test meal consisted of a toasted sandwich [white bread (75 g; Kingsmill; Allied Bakeries UK), Cheddar cheese (32.6 g) and butter (modified: 32.6 g; control: 29.4 g)], cornflakes (38 g, Kellogg's UK) served with UHT milk (195 g), and a strawberry milkshake [UHT milk (330 g) and strawberry sauce (19 g; Askeys; Silver Spoon Company UK)]. The lunch meal consisted of a toasted sandwich [white bread (60 g; Kingsmill; Allied Bakeries UK), Cheddar cheese (15 g) and butter (modified: 19.8 g; control: 18.6 g)] and a strawberry milkshake [UHT milk (modified: 352 g; control: 350 g) and strawberry sauce (27 g; Askeys; Silver Spoon Company UK)].

**FIGURE 3:**
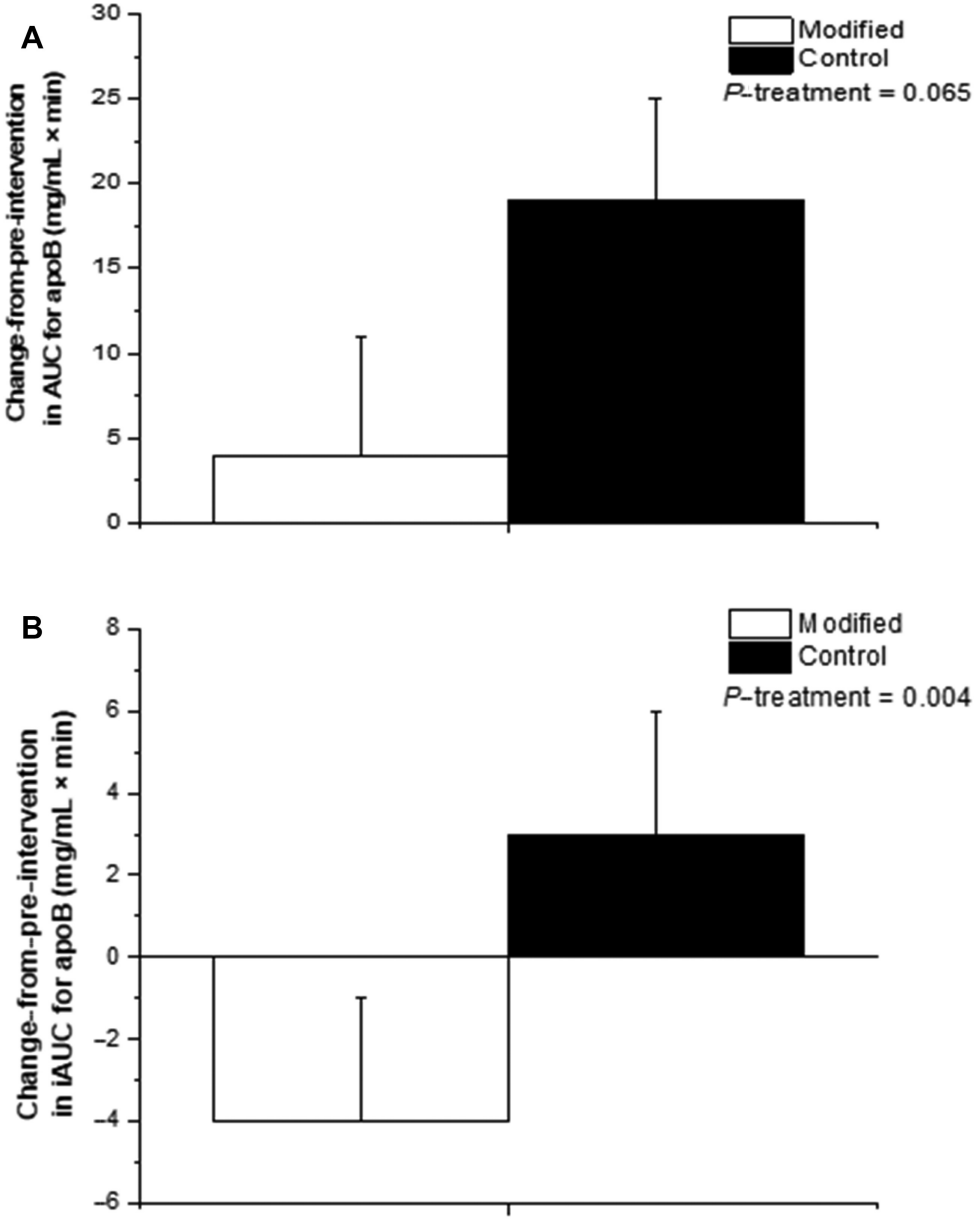
(A) ∆ AUC and (B) ∆ iAUC for the postprandial apoB response to test meals representative of the FA-modified and conventional dairy (control) diets consumed prior to and following the 12-wk interventions. Values are untransformed and unadjusted means ± SEMs, *n* = 47. Linear mixed-model analyses were used to calculate overall treatment effect based on Δ in each 12-wk dietary intervention (calculated by subtracting week 0 from week 12 values and week 20 from week 32 values), with adjustments made for fixed effects of baseline values of the assessed outcome measure at the beginning of each dietary period (i.e., the postprandial summary measure for the preintervention visit), period, treatment, sex, age, and BMI. Participant was included as a random effect. No period effects were observed in the model for any outcome measure. *P* ≤ 0.01 was deemed as significant to acknowledge multiplicity. FA, fatty acid; iAUC, incremental AUC; ∆, change from preintervention.

**TABLE 1:** Energy and nutrient content from the sequential test breakfast (0 min) and lunch meals (330 min) consumed by participants at baseline (week 0/week 20) and following 12-wk diets that incorporated the FA-modified and conventional (control) study products (week 12/week 32)^1^

	Modified			Control		
	Breakfast	Lunch	Total	Breakfast	Lunch	Total
Energy,^2^ MJ	4.3	2.6	6.9	4.1	2.5	6.6
Protein,^2^ g	36.1	20.9	57.0	39.7	19.6	59.3
Carbohydrate,^2^ g	105.9	64.6	170.5	101.4	63.3	164.7
Free sugars, g	16.5	22.3	38.8	15.0	21.5	36.5
Total fat,^2^ g	50.6	30.6	81.2	49.9	30.3	80.2
SFAs,^3^ g	24.5	14.8	39.3	31.7	19.1	50.8
MUFAs,^3^ g	20.0	12.1	32.1	12.3	7.4	19.7
TFAs,^3^ g	3.9	2.6	6.5	2.2	1.4	3.6
PUFAs,^3^ g	2.9	1.8	4.7	2.8	1.8	4.6

1 Values are total energy and macronutrient quantities of each test meal according to modified and control diet. Adapted from reference (8). FA, fatty acid; TFA, *trans* fatty acid.

2 Energy, protein, carbohydrate, and total fat content of the dairy product samples was measured in duplicate by SGS UK Ltd (Ealing, London).

3 Lipids extracted from the dairy product samples were analyzed in triplicate for FA composition by GC-flame ionization detection, as described elsewhere (19).

### Study visits

On the day preceding each study visit, participants were instructed to avoid alcohol and vigorous aerobic exercise. In addition, participants were provided with a standardized low-fat ready meal (<1.46 MJ; <7 g total fat) to consume on the evening before testing. During the night, and in the morning before study visit commencement, only low-nitrate water (Buxton Mineral Water; Nestlé Waters UK) could be consumed.

After a 12-h overnight fast, anthropometric assessments were conducted before a cannula was inserted into the forearm (antecubital vein) and a fasting blood sample was collected (−30 min). Fasting FMD was assessed, and a second fasting blood sample was drawn (0 min), before participants consumed a standardized test breakfast within 20 min. Postprandial blood draws were taken at 30, 60, 90, 120, 150, 180, 240, 300, 330, 360, 390, 420, and 480 min after the breakfast. Immediately after the 330-min blood sample had been collected, participants consumed a standardized test lunch meal within 15 min. Postprandial FMD was assessed directly after obtaining the 180-, 300-, and 420-min blood samples. No other food or drink, except for ad libitum low-nitrate water intake, was permitted throughout the study visit.

### Endothelial function assessment

Ultrasound assessment of FMD of the brachial artery was conducted on the uncannulated arm using a CX50 CompactXtreme Ultrasound System (Philips HealthCare, UK), in accordance with internationally accepted guidelines (21) and as described in detail elsewhere (5, 8). In brief, a single trained researcher performed all measurements for a given participant in a quiet, temperature-controlled clinical room (22° ± 1°C), which was darkened for endothelial function assessments. Scans were analyzed in a blinded manner using automated wall-tracking software (Vascular Research Tools 5; Medical Imaging Applications LLC). The %FMD response was calculated as the maximum percentage change in brachial artery diameter from baseline.

### Biochemical analyses

Blood samples were collected and processed, as described elsewhere (5, 8). Briefly, serum triacylglycerol (TG), nonesterified fatty acid (NEFA), glucose, and insulin concentrations were collected at all time points. Samples for serum apoB had similar time points, with the exclusion of 30, 90, and 330 min. At time points designated for FMD assessment (0, 180, 300, and 420 min), blood samples were also collected for quantification of plasma nitrite, nitrate, markers of endothelial activation, total lipid FA responses, and whole-blood culture for determination of LPS-stimulated cytokine production (see below).

Serum TG, apoB, NEFA, and glucose concentrations were analyzed with colorimetric assay kits (TG and glucose reagents: Instrumentation Laboratory Ltd; NEFA reagent: Alpha Laboratories Ltd) or an immunoturbidimetric assay (apoB reagent: Randox Laboratories Ltd) on an ILAB 600 autoanalyzer (Instrumentation Laboratory Ltd). Serum insulin concentrations were quantified by ELISA (Dako UK Ltd). Plasma concentrations of nitrite and nitrate were determined by HPLC (ENO-30; Eicom Corporation, USA) with online reduction of nitrate to nitrite and subsequent post-column derivatization with the Griess reagent (ENO-30 Analyzer; Eicom Corporation, USA) (5). Plasma concentrations of markers of endothelial activation, including soluble vascular cell adhesion molecule-1 (sVCAM-1), soluble intercellular adhesion molecule-1 (sICAM-1), E-selectin, and P-selectin were quantified by a Human Adhesion Molecule Magnetic Luminex Performance Assay 4-Plex kit (R&D Systems Europe Ltd) with xPONENT software 3.1 (Luminex, USA) on a Luminex 200 (Invitrogen, USA) instrument. Samples for each participant were analyzed within the same run or kit to prevent between-assay variation. Mean interassay CVs were <5% and <10% for automated assays (ILAB) and for all other analyses, respectively.

### LPS-stimulated cytokine analyses

For determination of whole-blood culture LPS-stimulated cytokine concentrations, uncentrifuged whole-blood samples collected in K2-EDTA tubes were diluted 1:9 with Roswell Park Memorial Institute 1640 medium (Sigma, UK) supplemented with 1% antibiotics, 1% l-glutamine, and 1% nonessential amino acids (BioScience, UK) (22). Subsequently, diluted blood samples were cultured in 12-well plates (Greiner Bio-one, UK) with 0.5 μg bacterial LPS/mL (*Escherichia coli* 026:B6; Sigma, UK), at a final concentration of 0.05 μg/mL. Cultures were incubated at 37°C for 24 h before centrifugation at 700 × *g* for 5 min at room temperature to isolate supernatant, which was stored at –20°C until analysis. A human cytokine premixed 5-Plex Panel (TNF-α, IL-6, IL-8, IL-1β, IL-10; R&D Systems Europe Ltd) was used to measure concentrations of cytokines in the whole-blood culture supernatant in a 1:2 dilution using a Luminex 200 with xPONENT software 3.1.

Measurement of monocyte count of each blood sample was performed by the Pathology Department at the Royal Berkshire Hospital (Reading, UK). Cytokine production was corrected for the number of monocytes in the whole-blood sample, expressed as mg × 10^3^ monocytes.

### Total lipid FA analyses

To assess changes in plasma FA status in response to our sequential 2-meal postprandial protocol, we measured the plasma total lipid FA pool. This pool is indicative of immediate FA intake and represents a mixture of all plasma lipid fractions that contain FA moieties, particularly cholesteryl esters, NEFAs, phospholipids, and TGs (23). Total lipids were extracted from K3-EDTA plasma samples isolated from blood samples collected at 0, 180, 300, and 420 min. Total plasma lipid was extracted using the method by Burdge et al. (24), and as described previously (8). In brief, FA methyl esters (FAMEs) were resolved on a 100-m fused silica capillary column (CP-SIL 88; Agilent Technologies, UK) using a gas chromatograph (Bruker 350; Bruker, Germany), with a flame ionization detector (25). Plasma FAMEs were identified based on retention time comparisons with an authentic standard (GLC #463; Nu-Chek-Prep, Inc., USA) and cross-referenced against published chromatograms (26). Carbon deficiency in the flame ionization detector response for FAMEs containing 4- to 10-carbon atoms was accounted for using a combined correction factor, which also converted FAMEs to FAs (27). Results were expressed as g/100 g total FAs.

### DNA extraction and genotyping

DNA was extracted from the buffy coat of whole fasted blood collected into K3-EDTA tubes using the Qiagen DNA Blood Mini Kit (Qiagen Ltd). Allelic discrimination of the *APOE* (*E2/E4,E2/E3,E2/E2,E3/E3,E3/E4*, or *E4/E4*; rs7412 and rs429358) and *eNOS* gene variants [Glu/Glu (GG), Glu/Asp (GT), and Asp/Asp (TT); rs1799983] were determined retrospectively using the Applied Biosystems RT-PCR 7300 instrument and Assay-on-Demand single nucleotide polymorphism genotyping assays (Life Technologies).

### Power calculation

The a priori–defined primary study outcome measure was change in the postprandial %FMD response. A minimum of 45 participants were required to complete both arms of the study to detect a 1.4%-unit (SD: 2.3) intergroup difference in the postprandial %FMD response, with 80% power and 5% significance. Fifty-two participants were recruited to allow for a 15% drop-out rate. A *P* value < 0.05 was considered significant for the primary outcome measure.

No formal sample-size calculations were performed for secondary outcomes of this study, which included the following: lipid [TGs, apoB, and NEFAs (*n* = 44–47)], glucose and insulin responses (*n* = 45–46), circulating biomarkers of endothelial activation and inflammation [nitrite, nitrate, sVCAM-1, sICAM-1, E-selectin, P-selectin; whole-blood culture LPS-stimulated cytokines (TNF-α, IL-6, IL-1β, IL-8, and IL-10) (*n* = 47–50)], and plasma total lipid FA responses (*n* = 47–49). The FAs deemed relevant to our dietary intervention (from 65 identified FAs) were selected for statistical analysis a priori (8). As discussed previously (5), in order to acknowledge the issue of multiplicity, a *P* value ≤ 0.01 was chosen a priori for assessment of significance of secondary outcome measures (28).

We also investigated the interactions between *APOE* and *eNOS* genotypes and dairy treatments on the primary and secondary outcomes (*n* = 39–51); these analyses were not powered, relied on retrospective genotyping, and therefore should be considered explorative.

### Statistical analyses

This was a proof-of-concept study designed to assess efficacy rather than a confirmatory trial to consider effectiveness (15); therefore, a per-protocol approach was adopted a priori (29). Results are given as untransformed and unadjusted means ± SEMs, unless otherwise stated.

Summary measures of postprandial responses, calculated using the trapezoidal rule, were expressed as AUC and incremental AUC (iAUC). The iAUC, calculated by subtracting the fasting value from all subsequent time-point values of the assessed outcome measure, indicated the specific responses to sequential meal ingestion, independent of fasting values (30). Due to the shape of the NEFA curve, AUC and iAUC were calculated from 120 min after the breakfast meal [the approximate time of NEFA minimum concentration (Cmin) after the first test meal] until 480 min postprandially (i.e., 360-min interval) (31). For variables with 10 (apoB) or 13 (TGs, NEFAs, glucose, and insulin) time points, analyses of maximum (peak) concentration reached after the test meals (Cmax) and time of maximum concentration (Tmax) were also assessed. Additional outcome measures for NEFAs included minimum concentration (Cmin), time to reach minimum concentration (Tmin), and percentage of NEFA suppression, which was calculated based on minimum concentration obtained before lunch (i.e., 30–330 min following the breakfast meal) (31). If data were missing for the AUC/iAUC calculations on a specific study visit, the missing time point was imputed by taking the mean of the surrounding values. If time points were missing for Cmax, Tmax, Cmin, and Tmin assessment, the chosen concentration or time was based on available data from the study visit. A participant's study visit data were excluded from analysis if the fasting time point was missing or if a large proportion of time-point data were missing (i.e., ≥40% data and/or 3 consecutive time points).

Statistical analyses were performed with the use of SAS 9.4 University edition statistical software (SAS Institute Inc.) and IBM SPSS Statistics 25.0 (Statistical Product and Service Solutions; IBM Corp.). Logarithmic transformations were performed on all variables where the assumption of normality did not hold. Baseline characteristics of participants randomly assigned to consume the modified or conventional (control) dairy diet at week 0 (i.e., during their first dietary exchange period) and between genotype groups (*APOE* and *eNOS*) were compared by independent *t* tests and chi-square tests for continuous and categorical variables, respectively. For all primary and secondary outcome measures, linear mixed-model analyses (PROC MIXED; SAS Institute Inc.) were used to calculate overall treatment effects with change from preintervention (Δ) in postprandial summary measures for each 12-wk dietary intervention (calculated by subtracting week 0 from week 12 values and week 20 from week 32 values) as the dependent variable, adjusted for the fixed effects of baseline values of the assessed outcome measure at the beginning of each dietary period (i.e., the postprandial summary measure for the preintervention visit), period, treatment, sex, age, and BMI. Fixed-effect covariates were retained in all linear mixed models regardless of their degrees of significance. Participant was included as a random effect. In additional analyses, the interactive effects of *APOE* and *eNOS* genotype groups and treatment based on Δ in postprandial summary measures for each 12-wk dietary intervention were also assessed by using linear mixed models, with the addition of genotype and genotype × treatment fixed effects in the overall treatment effect model described above. Other than transforming variables to improve normality, modeling assumptions were not validated in a formal way. Linear mixed models are generally robust to some misspecifications (32).

## Results

### Baseline characteristics

A total of 52 participants successfully completed the study (see **Supplemental Figure 1** for flowchart). Reasons cited for study withdrawal included the following: unable to comply with the intervention (*n* = 8), time commitment (*n* = 8), health or personal issues unrelated to the intervention (*n* = 5), and unsuitability for cannulation (*n* = 3). There were no large biases in the age and sex of participants who were randomly assigned (*n* = 76) and those who completed the intervention (*n* = 52).

Data from 4 participants were not analyzed or were excluded from FMD analysis due to poor image quality or technical issues during recording of ultrasound measurements. Due to technical difficulties with cannulation at 3 out of 4 visits, 1 participant was fully excluded from analyses of all blood variables. Baseline characteristics of participants who completed the study are presented in **Table 2**; there were no significant differences in the baseline characteristics of participants randomly allocated to consume the FA-modified or conventional (control) dietary intervention period during their first dietary exchange period (week 0). The breakdown of the ethnicity of the cohort was as follows: Asian, 4% (*n*  =  2); Black, 4% (*n*  =  2); Chinese/Far Eastern, 4% (*n*  =  2); and White, 88% (*n*  =  46). Relative to preintervention (baseline), there was no significant treatment effect for body mass or waist circumference between dairy diets (15). Similarly, physical activity scores (as assessed by the IPAQ) did not significantly differ between treatments, relative to baseline (data not shown). The dairy diets and test meals were well tolerated by the participants in both treatment arms.

**TABLE 2:** Baseline characteristics of participants randomly assigned to consume FA-modified and conventional (control) dairy products during their first dietary exchange period^1^

Characteristics	Overall group (*n* = 52)	Modified (*n* = 22)	Control (*n* = 30)
Sex (M/F), *n*/*n*	31/21	15/7	16/14
Age, y	53 ± 2	51 ± 3	54 ± 2
Body mass, kg	77.5 ± 1.9	78.6 ± 2.8	76.7 ± 2.6
BMI, kg/m^2^	26.0 ± 0.4	26.1 ± 0.7	25.0 ± 0.6
Waist circumference, cm	90.6 ± 1.4	91.5 ± 2.1	86.7 ± 1.9
SBP, mm Hg	121 ± 2	120 ± 3	121 ± 2
DBP, mm Hg	70 ± 1	70 ± 2	70 ± 1
Fasting serum biomarkers			
TC, mmol/L	5.71 ± 0.14	5.62 ± 0.20	5.77 ± 0.20
LDL-C, mmol/L	3.48 ± 0.11	3.43 ± 0.16	3.52 ± 0.15
HDL-C, mmol/L	1.58 ± 0.04	1.53 ± 0.07	1.62 ± 0.05
TG, mmol/L	1.18 ± 0.06	1.21 ± 0.09	1.15 ± 0.09
Glucose, mmol/L	5.53 ± 0.11	5.64 ± 0.21	5.44 ± 0.10
Insulin, pmol/L	36.4 ± 2.9	35.3 ± 4.0	37.1 ± 4.0
HOMA-IR	1.44 ± 0.12	1.42 ± 0.17	1.46 ± 0.16
CVD risk score^2^	3.0 ± 0.2	2.8 ± 0.2	3.2 ± 0.3

1 Values are unadjusted means ± SEMs or *n; n* = 52 (overall group). No significant differences between participants randomly assigned to consume the modified and control dairy products during their first dietary exchange period were observed for any of the baseline characteristics using independent *t* tests and chi-square test for continuous and categorical variables, respectively (*P* > 0.01). CVD, cardiovascular disease; DBP, diastolic blood pressure; FA, fatty acid; LDL-C, LDL cholesterol; HDL-C, HDL cholesterol; RESET, REplacement of SaturatEd fat in dairy on Total cholesterol; SBP, systolic blood pressure; TC, total cholesterol; TG, triacylglycerol.

2 Assessed with the use of a modified Framingham risk score, where a score of ≥2 points relates to a 50% higher risk of CVD than the population mean (15).

### Postprandial endothelial function response

**FIGURE 2:**
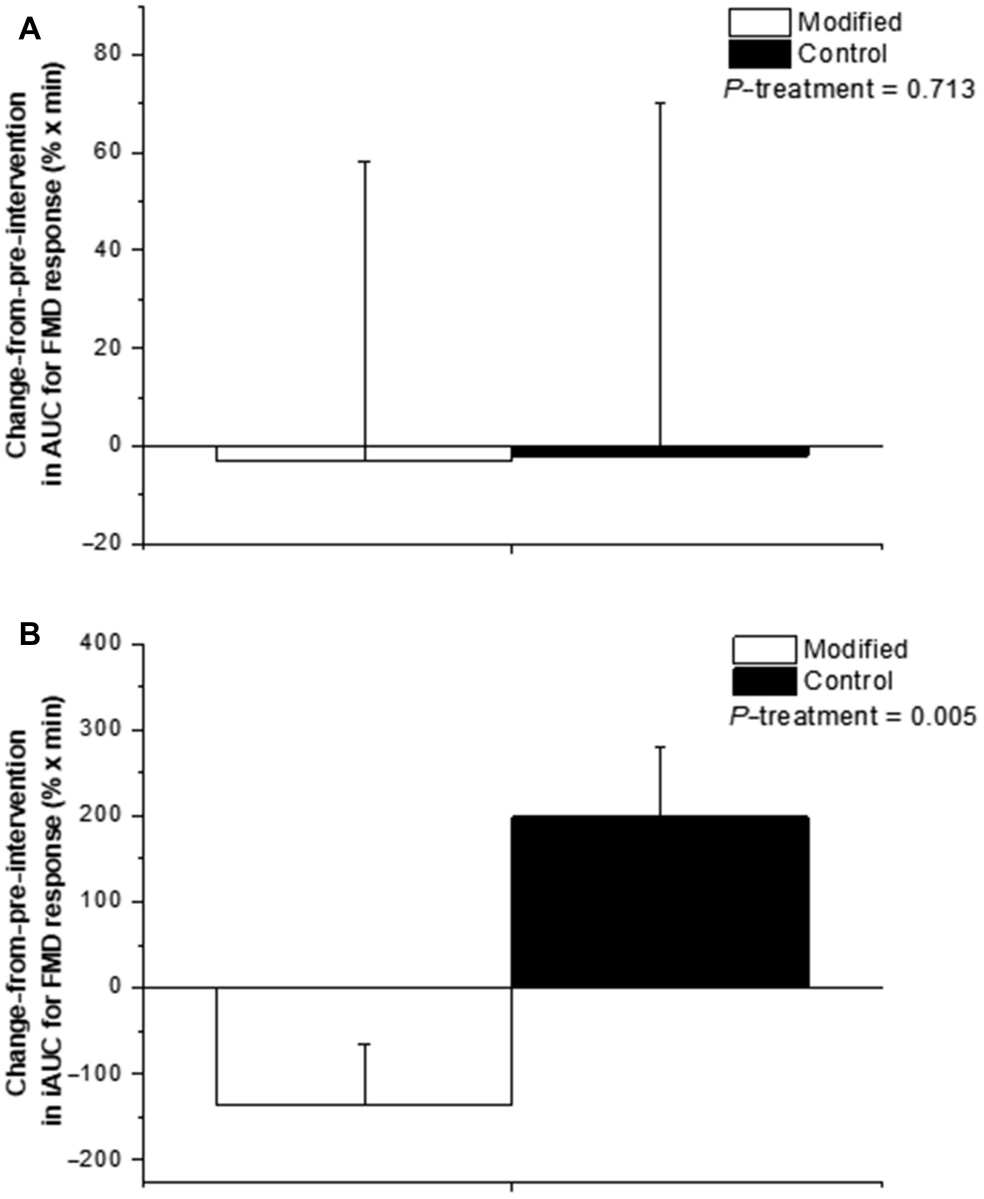
(A) ∆ AUC and (B) ∆ iAUC for the postprandial %FMD response to test meals representative of the FA-modified and conventional dairy (control) diets consumed prior to and following the 12-wk interventions. Values are untransformed and unadjusted means ± SEMs, *n* = 48. Linear mixed-model analyses were used to calculate overall treatment effect based on Δ in each 12-wk dietary intervention (calculated by subtracting week 0 from week 12 values and week 20 from week 32 values), with adjustments made for fixed effects of baseline values of the assessed outcome measure at the beginning of each dietary period (i.e., the postprandial summary measure for the preintervention visit), period, treatment, sex, age, and BMI. Participant was included as a random effect. No period effects were observed in the model for any outcome measure. *P* < 0.05 was deemed as significant for the primary outcome. FA, fatty acid; FMD, flow-mediated dilatation; iAUC, incremental AUC; %FMD, percentage of flow-mediated dilatation; ∆, change from preintervention.

In line with our previously reported RESET chronic study findings (*n* = 50) (5), here we found that fasting endothelial function (as assessed by %FMD response) increased relative to baseline after the FA-modified diet (+0.31 ± 0.15%), yet decreased after the control diet (–0.49 ± 0.16%; *P* = 0.0005; *n* = 48). The AUC and iAUC for the %FMD response are presented in **Figure 2**A and B, respectively. Relative to preintervention, there was a differential impact of the dairy treatment on the ∆ iAUC (but not AUC) for the %FMD response, with an increase (+199 ± 82% × min) observed following the conventional treatment compared with a decrease (–135 ± 69% × min) following the FA-modified dairy treatment (*P* = 0.005) (Figure 2B).

### Postprandial lipid, glucose, and insulin responses

The postprandial summary measures of lipid, glucose, and insulin responses are presented in **Table 3**. The AUC and iAUC for the apoB response are presented in **Figure 3**A and B, respectively. A differential effect was observed for the ∆ iAUC (but not AUC or other postprandial summary responses) for the apoB response relative to preintervention, with a decrease (–4 ± 3 mg/mL × min) observed following the FA-modified treatment compared with an increase (+3 ± 3 mg/mL × min) after the control dairy treatment (*P* = 0.004; Figure 3B). There was no differential impact of the 2 dairy treatments on the postprandial summary measures for the TG, NEFA, glucose, and insulin responses.

**TABLE 3:** Postprandial summary measures for serum lipid, glucose, and insulin responses to sequential high-fat mixed-meal challenges representative of the FA-modified and conventional (control) dairy diets consumed preintervention (week 0/week 20) and postintervention (week 12/week 32), and the Δ following each 12-wk dietary intervention in adults with moderate CVD risk^1^

	Modified diet and test meal			Control diet and test meal			
	Preintervention	Postintervention	Δ	Preintervention	Postintervention	Δ	*P* ^2^
TGs							
Cmax, mmol/L	2.84 ± 0.15	2.93 ± 0.18	0.12 ± 0.15	2.58 ± 0.12	2.57 ± 0.14	0.06 ± 0.11	0.620
Tmax, min	359 ± 9	330 ± 10	−25 ± 11	334 ± 9	324 ± 12	−10 ± 25	0.429
AUC,^3^ mmol/L × min	953 ± 52	1018 ± 72	72 ± 55	879 ± 41	906 ± 54	55 ± 40	0.260
iAUC,^3^ mmol/L × min^3^	354 ± 25	388 ± 30	34 ± 28	321 ± 23	332 ± 27	19 ± 20	0.138
apoB							
Cmax, g/mL	1.04 ± 0.03	1.05 ± 0.03	0.01 ± 0.02	1.03 ± 0.03	1.07 ± 0.03	0.04 ± 0.02	0.293
Tmax, min	209 ± 23	208 ± 22	−1 ± 31	210 ± 22	224 ± 23	14 ± 29	0.360
NEFAs							
Cmin_30–330_,^3^ μmol/L	113 ± 6	111 ± 6	−3 ± 6	109 ± 5	118 ± 6	11 ± 6	0.054
Tmin_30–330_,^3^ min	139 ± 6	129 ± 5	−10 ± 7	134 ± 7	131 ± 6	−3 ± 7	0.884
Suppression_30–330_,^3^ %	79 ± 1	77 ± 1	2 ± 1	78 ± 2	75 ± 1	4 ± 2	0.147
Cmax_120–480_,^3^ μmol/L	442 ± 19	452 ± 21	4 ± 22	482 ± 20	458 ± 17	−25 ± 24	0.721
Tmax_120–480_, min	361 ± 5	322 ± 15	−42 ± 17	358 ± 6	354 ± 5	−3 ± 7	0.083
AUC_120–480_,^3^ mmol/L × min	92 ± 4	95 ± 4	2 ± 4	97 ± 4	97 ± 3	1 ± 3	0.509
iAUC_120–480_,^3^ mmol/L × min	46 ± 3	50 ± 4	4 ± 4	52 ± 4	50 ± 3	-3 ± 4	0.553
Glucose							
Cmax,^3^ mmol/L	8.25 ± 0.21	8.38 ± 0.20	–0.10 ± 0.22	8.02 ± 0.20	8.07 ± 0.22	0.06 ± 0.16	0.221
Tmax, min	223 ± 24	258 ± 25	31 ± 27	251 ± 24	287 ± 23	16 ± 25	0.900
AUC,^3^ mmol/L × min	2937 ± 61	2925 ± 63	–15 ± 32	2866 ± 59	2888 ± 85	44 ± 42	0.425
iAUC, mmol/L × min	345 ± 39	358 ± 49	14 ± 50	263 ± 42	302 ± 38	53 ± 42	0.613
Insulin							
Cmax, pmol/L	560 ± 49	587 ± 55	26 ± 23	515 ± 38	501 ± 28	–18 ± 27	0.303
Tmax, min	165 ± 24	203 ± 24	20 ± 30	227 ± 24	171 ± 23	–63 ± 25	0.222
AUC,^3^ µmol/L × min	122 ± 10	120 ± 12	–1 ± 6	114 ± 8	111 ± 7	–1 ± 3	0.592
iAUC,^3^ µmol/L × min	102 ± 9	101 ± 11	0 ± 6	95 ± 7	89 ± 6	–5 ± 4	0.326

1 Values are untransformed and unadjusted means ± SEMs. For all variables, *n* = 46; except for apoB, *n* = 47; NEFAs, *n* = 44; and insulin, *n* = 45. Time interval for AUC and iAUC: 480 min for all variables, except for 360 min for NEFAs. Cmax, maximum concentration; Cmin, minimum concentration; CVD, cardiovascular disease; FA, fatty acid; iAUC, incremental AUC; NEFA, nonesterified fatty acid; TG, triacylglycerol; Tmax, time to reach maximum concentration; Tmin, time to reach minimum concentration; Δ, change from preintervention.

2 Linear mixed-model analyses were used to calculate overall treatment effect based on Δ in each 12-wk dietary intervention (calculated by subtracting week 0 from week 12 values and week 20 from week 32 values), with adjustments made for fixed effects of baseline values of the assessed outcome measure at the beginning of each dietary period (i.e., the postprandial summary measure for the preintervention visit), period, treatment, sex, age, and BMI. Participant was included as a random effect. No period effects were observed in the model for any outcome measure. For all outcome measures, *P* ≤ 0.01 was deemed as significant to acknowledge multiplicity.

3 Indicates data were log transformed prior to analysis.

### Postprandial response for circulating markers of endothelial activation and inflammation

The FA composition of the dairy treatment had no effect on the ∆ postprandial endothelial activation or whole-blood culture LPS-stimulated cytokine summary measures (**Table 4**). However, there was a tendency for an increase (+10.9 ± 6.8 μmol/L × min) in the ∆ iAUC for the postprandial nitrite response following the conventional dairy treatment, compared with a decrease following the FA-modified treatment (–6.4 ± 5.0 μmol/L × min; *P* = 0.027).

**TABLE 4:** Postprandial summary measures for endothelial function and circulating biomarkers of endothelial activation and inflammatory responses to sequential high-fat, mixed-meal challenges representative of the FA-modified and conventional (control) dairy diets consumed preintervention (week 0/week 20) and postintervention (week 12/week 32), and the Δ following each 12-wk dietary intervention in adults with moderate CVD risk^1^

	Modified diet and test meal			Control diet and test meal			
	Preintervention	Postintervention	Δ	Preintervention	Postintervention	Δ	*P* ^2^
Plasma nitrite,^3^ μmol/L × min							
AUC	63.8 ± 7.8	64.5 ± 8.2	0.6 ± 5.3	65.5 ± 59.8	59.8 ± 10.4	–4.4 ± 8.1	0.552
iAUC	4.2 ± 4.8	–2.2 ± 2.5	–6.4 ± 5.0	–0.8 ± 4.7	10.1 ± 5.1	10.9 ± 6.8	0.027
Plasma nitrate,^3^ μmol/L × min							
AUC	5715 ± 517	5679 ± 329	–36 ± 400	5288 ± 271	5671 ± 461	489 ± 466	0.352
iAUC	–1656 ± 302	–1734 ± 338	–78 ± 379	–1703 ± 248	–1160 ± 311	510 ± 370	0.086
Adhesion molecules,^3^ ng/mL × min							
Plasma sVCAM-1							
AUC	225.9 ± 15.3	224.3 ± 15.0	–1.6 ± 6.1	236.6 ± 16.2	222.2 ± 16.6	–18.8 ± 9.9	0.134
iAUC	–0.8 ± 4.2	–2.6 ± 5.4	–1.8 ± 7.9	3.7 ± 5.6	3.2 ± 3.1	–0.5 ± 6.2	0.286
Plasma sICAM-1							
AUC	37.7 ± 3.7	35.7 ± 3.3	–2.0 ± 1.5	36.4 ± 3.8	37.6 ± 4.0	1.2 ± 1.6	0.407
iAUC	0.7 ± 1.5	1.0 ± 1.7	0.3 ± 2.5	1.0 ± 1.8	–0.9 ± 1.7	–1.9 ± 2.7	0.329
Plasma E-selectin							
AUC	10.3 ± 1.0	10.5 ± 0.9	0.1 ± 0.2	10.3 ± 0.9	10.7 ± 1.0	0.2 ± 0.3	0.841
iAUC^3^	–0.3 ± 0.2	–0.3 ± 0.2	0.0 ± 0.3	–0.3 ± 0.2	–0.2 ± 0.2	0.1 ± 0.2	0.618
Plasma P-selectin							
AUC	10.7 ± 0.7	10.9 ± 0.8	0.2 ± 0.2	10.7 ± 0.7	11.1 ± 0.8	0.4 ± 0.3	0.845
iAUC	0.0 ± 0.2	–0.4 ± 0.2	–0.4 ± 0.4	–0.5 ± 0.2	0.1 ± 0.2	0.6 ± 0.3	0.340
Whole-blood culture LPS-stimulated cytokines, mg × 10^3^ monocytes × min							
TNF-α							
AUC^3^	4.94 ± 0.33	5.41 ± 0.30	0.47 ± 0.22	4.99 ± 0.29	5.49 ± 0.30	0.60 ± 0.35	0.858
iAUC	–0.17 ± 0.20	–0.19 ± 0.21	–0.02 ± 0.28	0.16 ± 0.24	–0.26 ± 0.16	–0.41 ± 0.26	0.854
IL-6							
AUC^3^	32.8 ± 1.7	35.4 ± 2.0	2.6 ± 1.0	33.0 ± 1.7	35.0 ± 1.8	2.0 ± 1.6	0.474
iAUC	–2.15 ± 0.98	–2.14 ± 1.08	0.01 ± 1.58	0.47 ± 1.31	–2.66 ± 0.87	–3.13 ± 1.48	0.941
IL-1β							
AUC^3^	11.8 ± 0.6	12.0 ± 0.6	0.2 ± 0.4	12.5 ± 0.6	13.5 ± 0.7	1.2 ± 0.7	0.138
iAUC	0.32 ± 0.35	0.52 ± 0.33	0.20 ± 0.50	1.50 ± 0.36	1.24 ± 0.41	–0.23 ± 0.57	0.050
IL-8							
AUC	48.9 ± 3.9	54.1 ± 5.7	3.9 ± 4.5	51.2 ± 5.8	48.9 ± 5.6	–1.1 ± 4.9	0.134
iAUC	–4.32 ± 2.45	–2.91 ± 3.26	1.41 ± 3.34	–3.40 ± 4.17	–6.92 ± 2.64	–3.52 ± 3.37	0.150
IL-10							
AUC	0.29 ± 0.02	0.30 ± 0.03	0.01 ± 0.02	0.35 ± 0.04	0.31 ± 0.03	–0.03 ± 0.03	0.234
iAUC	–0.06 ± 0.02	–0.06 ± 0.02	–0.01 ± 0.03	–0.03 ± 0.02	–0.08 ± 0.02	–0.05 ± 0.03	0.855

1 Values are untransformed and unadjusted means ± SEMs. For all variables, *n* = 50; except for TNF-α, IL-1β, IL-6, IL-10, *n* = 49, and IL-8, *n* = 47. The time interval for AUC and iAUC: 420 min for all variables. CVD, cardiovascular disease; FA, fatty acid; iAUC, incremental AUC; sICAM-1, soluble intercellular adhesion molecule-1; sVCAM-1, soluble vascular adhesion molecule-1; Δ, change from preintervention.

2 Linear mixed-model analyses were used to calculate overall treatment effect based on Δ in each 12-wk dietary intervention (calculated by subtracting week 0 from week 12 values and week 20 from week 32 values), with adjustments made for fixed effects of baseline values of the assessed outcome measure at the beginning of each dietary period (i.e., the postprandial summary measure for the preintervention visit), period, treatment, sex, age, and BMI. Participant was included as a random effect. No period effects were observed in the model for any outcome measure. For all outcome measures, *P* ≤ 0.01 was deemed significant to acknowledge multiplicity.

3 Indicates data were log transformed prior to analysis.

### Postprandial plasma total lipid FA responses

Differential effects were evident for the postprandial plasma total lipid FA responses (∆ AUC and iAUC) following the 2 dairy treatments (see **Table 5**). AUC, which included the fasting value, indicated that, relative to preintervention, the ∆ abundance of 16:0 (palmitic acid) and total SFAs was significantly higher following consumption of the conventional dairy treatment (12-wk diet and representative test meals), relative to the FA-modified dairy treatment (both *P* = 0.001). The ∆ AUC for total *cis*-18:1 [predominantly *cis*-9 18:1 (oleic acid)], total *cis*-MUFAs, total *trans*-18:1 [including *trans*-9 (elaidic acid) and *trans*-10 (octadecenoic acid)], total *trans*-MUFAs, and *trans* fatty acids (TFAs) increased after consuming the FA-modified treatment compared with the conventional dairy treatment (all *P* < 0.0001). The proportion of *trans*-11 18:1 (vaccenic acid) in the postprandial plasma lipid pool was similar following FA-modified and control dairy treatments (*P* > 0.01 for the ∆ AUC). Relative to the conventional dairy treatment, the ∆ AUC was increased for total conjugated linoleic acids (CLAs) and decreased for n–3 PUFAs following the 12-wk modified dairy diet and test meals (*P* < 0.0001 and *P* = 0.005, respectively).

**TABLE 5:** Postprandial summary measures for selected plasma total lipid FA responses to sequential high-fat, mixed-meal challenges representative of the FA-modified and conventional (control) dairy diets consumed preintervention (week 0/week 20) and postintervention (week 12/week 32), and the Δ following each 12-wk dietary intervention in adults with moderate CVD risk^1^

	Modified diet and test meal			Control diet and test meal			
FAs, g/100 g total FAs × min	Preintervention	Postintervention	Δ	Preintervention	Postintervention	Δ	*P* ^2^
SFAs							
12:0							
AUC	48 ± 7	39 ± 5	–8 ± 5	67 ± 13	74 ± 11	3 ± 10	0.038
iAUC	8 ± 7	5 ± 6	–2 ± 5	28 ± 14	33 ± 11	2 ± 10	0.027
14:0							
AUC	694 ± 24	729 ± 23	40 ± 23	839 ± 28	888 ± 31	46 ± 28	0.073
iAUC	282 ± 15	301 ± 15	20 ± 13	433 ± 24	407 ± 20	–27 ± 18	0.009
15:0							
AUC	119 ± 2	127 ± 2	8 ± 2	138 ± 3	152 ± 3	13 ± 2	0.123
iAUC	22 ± 1	23 ± 1	2 ± 1	39 ± 2	37 ± 2	–2 ± 2	0.313
16:0							
AUC	9755 ± 91	9673 ± 95	–72 ± 64	10,358 ± 73	10,561 ± 105	200 ± 77	0.001
iAUC	142 ± 33	194 ± 36	51 ± 44	837 ± 48	791 ± 51	–43 ± 63	<0.0001
17:0							
AUC	117 ± 3	118 ± 3	1 ± 3	128 ± 3	131 ± 4	3 ± 4	0.221
iAUC	1 ± 3	3 ± 3	2 ± 3	12 ± 4	8 ± 4	–4 ± 3	0.580
18:0							
AUC	3406 ± 42	3412 ± 44	6 ± 27	3262 ± 45	3218 ± 43	–49 ± 45	0.036
iAUC	255 ± 38	282 ± 45	24 ± 51	181 ± 43	118 ± 42	–67 ± 55	0.033
Total SFAs^3,4^							
AUC	14,727 ± 126	14,698 ± 127	–14 ± 96	15,400 ± 111	15,674 ± 145	259 ± 135	0.001
iAUC	741 ± 69	838 ± 78	96 ± 88	1579 ± 109	1461 ± 94	–114 ± 125	0.003
MUFAs							
*cis*-9 18:1^3^							
AUC	9356 ± 129	9665 ± 128	500 ± 218	8732 ± 117	8689 ± 121	–62 ± 82	<0.0001
iAUC	194 ± 68	237 ± 64	50 ± 84	–323 ± 54	–255 ± 47	76 ± 72	<0.0001
Total *cis-*18:1^3,5^							
AUC	10,086 ± 133	10,416 ± 132	536 ± 232	9457 ± 124	9405 ± 127	–243 ± 355	<0.0001
iAUC	204 ± 63	225 ± 66	28 ± 81	–353 ± 56	–281 ± 48	80 ± 76	<0.0001
Total *cis*-MUFAs^3,6^							
AUC	10,850 ± 150	11,203 ± 150	369 ± 146	10,258 ± 139	10,260 ± 137	–208 ± 384	<0.001
iAUC	272 ± 67	306 ± 73	41 ± 88	–238 ± 59	–158 ± 57	90 ± 82	<0.0001
*trans*-9 18:1							
AUC	85 ± 3	108 ± 3	23 ± 4	53 ± 2	49 ± 2	–5 ± 3	<0.0001
iAUC	33 ± 5	45 ± 5	11 ± 5	4 ± 4	–2 ± 4	–7 ± 3	<0.0001
*trans*-10 18:1^3^							
AUC	222 ± 9	276 ± 8	59 ± 8	57 ± 2	52 ± 2	–5 ± 2	<0.0001
iAUC	184 ± 9	174 ± 8	–10 ± 8	16 ± 3	11 ± 3	–5 ± 3	<0.001
*trans*-11 18:1							
AUC	100 ± 3	108 ± 4	10 ± 4	102 ± 2	102 ± 3	–2 ± 4	0.146
iAUC	41 ± 3	42 ± 4	1 ± 4	41 ± 3	34 ± 2	–8 ± 3	0.013
Total *trans*-18:1^3,7^							
AUC	657 ± 19	811 ± 20	167 ± 23	358 ± 7	362 ± 9	–4 ± 13	<0.001
iAUC	422 ± 22	430 ± 22	8 ± 19	118 ± 12	101 ± 11	–17 ± 12	<0.0001
Total *trans*-MUFAs^3,8^							
AUC	855 ± 21	1017 ± 22	162 ± 21	552 ± 9	564 ± 10	2 ± 16	<0.0001
iAUC	424 ± 22	435 ± 23	12 ± 22	122 ± 13	106 ± 12	–18 ± 13	<0.0001
Total TFAs^3,9^							
AUC	1006 ± 23	1191 ± 25	186 ± 23	695 ± 11	718 ± 12	23 ± 14	<0.0001
iAUC	444 ± 23	455 ± 24	13 ± 23	130 ± 15	113 ± 14	–19 ± 15	<0.0001
PUFAs							
Total CLAs^3,10^							
AUC	118 ± 4	151 ± 6	33 ± 4	108 ± 4	117 ± 4	5 ± 5	<0.0001
iAUC	39 ± 3	36 ± 3	−3 ± 2	25 ± 2	21 ± 2	–4 ± 1	0.005
Total n–3 PUFAs^3,11^							
AUC	1458 ± 50	1330 ± 43	–129 ± 40	1482 ± 52	1415 ± 41	–67 ± 40	0.005
iAUC	–122 ± 19	–148 ± 12	–27 ± 21	–157 ± 25	–127 ± 13	28 ± 24	0.02
Total n–6 PUFAs^3,12^							
AUC	13,431 ± 241	13,027 ± 248	–436 ± 203	13,640 ± 216	13,389 ± 235	–208 ± 146	0.119
iAUC	–1336 ± 110	–1438 ± 105	–109 ± 109	–1300 ± 122	–1263 ± 110	25 ± 122	0.069

1 Values are untransformed and unadjusted means ± SEMs. For all variables, *n* = 47–49. The time interval for AUC and iAUC: 420 min for all variables. CLA, conjugated linoleic acid; CVD, cardiovascular disease; FA, fatty acid; iAUC, incremental AUC; TFA, *trans* fatty acid; Δ, change from preintervention.

2 Linear mixed-model analyses were used to calculate overall treatment effect based on Δ in each 12-wk dietary intervention (calculated by subtracting week 0 from week 12 values and week 20 from week 32 values), with adjustments made for fixed effects of baseline values of the assessed outcome measure at the beginning of each dietary period (i.e., the postprandial summary measure for the preintervention visit), period, treatment, sex, age, and BMI. Participant was included as a random effect. No period effects were observed in the model for any outcome measure. For all outcome measures, *P* ≤ 0.01 was deemed significant to acknowledge multiplicity.

3 Indicates data were log-transformed prior to analysis.

4 Includes 6:0, 7:0, 8:0, 9:0, 10:0, 11:0, 12:0, 13:0 iso, 13:0 anteiso, 13:0, 14:0 iso, 14:0, 15:0 anteiso, 15:0, 16:0 iso, 16:0, 17:0 iso, 17:0 anteiso, 17:0, 18:0 iso, 18:0, 19:0, 20:0, 22:0, and 24:0.

5 Includes *cis*-9 18:1, *cis*-11 18:1, *cis*-12 18:1, *cis*-13 18:1, *cis*-14 18:1, *cis*-15 18:1, and *cis*-16 18:1.

6 Includes *cis*-9 10:1, *cis*-10 11:1, *cis*-9 12:1, 13:1 (unknown bond position), *cis*-9 14:1, *cis*-10 15:1, *cis*-9 16:1, *cis*-13 16:1, *cis*-10 17:1, *cis*-9 17:1, *cis*-9 18:1, *cis*-11 18:1, *cis*-12 18:1, *cis*-13 18:1, *cis*-14 18:1 *cis*-15 18:1, *cis*-16 18:1, 19:1 (unknown bond position), *cis*-5 20:1, *cis*-8 20:1, *cis*-11 20:1, *cis*-13 22:1, and *cis*-15 24:1.

7 Includes *trans*-4 18:1, *trans*-6 18:1, *trans*-7 18:1, *trans*-8 18:1, *trans*-9 18:1, *trans*-10 18:1, *trans*-11 18:1, *trans*-12 18:1, *trans*-15 18:1, and *trans*-16 18:1.

8 Includes *trans*-9 14:1, *trans*-9 16:1, *trans*-11 16:1, *trans*-13 16:1, *trans*-10 17:1, *trans*-4 18:1, *trans*-6 18:1, *trans*-7 18:1, *trans*-8 18:1, *trans*-9 18:1, *trans*-10 18:1, *trans*-11 18:1, *trans*-12 18:1, *trans*-15 18:1, and *trans*-16 18:1.

9 Includes *trans*-18:1, *trans*-11, 15 18:2, *trans*-9, 12 18:2, *cis*-9, *trans*-13 18:2, *cis*-10, *trans*-14 18:2, *cis*-9, *trans*-12 18:2, *trans*-9, 12 18:2, *trans*-11, *cis*-15 18:2, and *trans*-12, *cis*-15 18:2.

10 Includes a peak that contains mainly *cis*-9, *trans*-11 CLA, but also *trans*-7, *cis*-9 CLA, *trans*-8, *cis*-10 CLA, and *trans*-6, *cis*-8 CLA.

11 Includes *trans*-11, 15 18:2, *trans*-11, *cis*-15 18:2, *trans*-12, *cis*-15 18:2, *cis*-9, 12, 15, 18:3, *cis*-11, 14, 17 20:3, *cis*-5, 8, 11, 14, 17 20:5, *cis*-7, 10, 13, 16, 19 22:5, and *cis*-4, 7, 10, 13, 16, 19 22:6.

12 Includes *trans*-9, 12 18:2, *cis-9, trans*-12 18:2, *trans*-9, *cis*-12 18:2, *cis*-9, 12 18:2, *cis*-6, 9, 12 18:3, *cis*-11, 14 20:2, *cis*-8, 11, 14 20:3, *cis*-5, 8, 11, 14 20:4, *cis*-13, 16 20:2, and *cis*-7, 10, 13, 16 22:4.

For the ∆ iAUC (which estimates the specific response to the test meals), there was a decrease found for 14:0 (myristic acid) and total SFAs after the conventional dairy treatment (12-wk dietary intervention and representative test meals) compared with the FA-modified treatment (*P* = 0.009 and *P* = 0.003, respectively). The ∆ iAUC for palmitic acid was higher following the FA-modified treatment compared with the conventional dairy treatment (*P* < 0.0001). The ∆ iAUC for total *cis*-18:1, oleic acid, and total *cis*-MUFAs was significantly increased following the conventional dairy diet and tests meals relative to the FA-modified dairy treatment (*P* < 0.0001). After the 12-wk diet, compared with preintervention, consumption of the FA-modified test meals led to an increase in the iAUC for the abundance of elaidic acid, relative to a decrease following the control dairy test meals (*P* < 0.0001). The ∆ iAUC for total *trans*-18:1, *trans*-MUFAs, and TFAs was decreased following the conventional dairy diet and representative test meals compared with the modified dairy treatment (all *P* < 0.0001). The ∆ AUC for octadecenoic acid was lower after the FA-modified treatment compared with the control dairy diet and test meals (*P* < 0.001), with a similar AUC response for vaccenic acid between treatments. Relative to the FA-modified diet and test meals, the ∆ iAUC was lower for total CLAs following the control dairy treatment (*P* = 0.005).

### Postprandial responses according to *APOE* and *eNOS* genotype groups

When genotyped retrospectively for *APOE*, 44 of the 52 participants were included in the analyses (**Supplemental Table 1**). Of these, *n* = 29 were identified as the wild-type homozygous *E3/E3* group and *n* = 15 as *E4* carriers [*E3/E4* (*n* = 14) and *E4/E4* (*n* = 1)]. Since a low number of participants were identified as *E2/E3* (*n* = 6) and *E2/E4* (*n* = 2) genotype groups, they were excluded from larger datasets and *E4* carrier groups (*E3/E4* and *E4/E4*) were pooled for analysis.

For the *eNOS* polymorphism, 23 of the 52 participants who participated in the study were identified as Glu298 homozygotes (GG), 24 as Glu298Asp heterozygotes (GT), and 5 as Asp298 homozygotes (TT) (**Supplemental Table 2**). Asp298 carriers (GT and TT groups) were combined for analysis because the TT genotype is relatively rare.

No significant differences in baseline characteristics were observed between the *APOE* or *eNOS* genotype groups (Supplemental Tables 1 and 2). There was no influence of *APOE* or *eNOS* genotype on the ∆ AUC or iAUC for the %FMD response and no significant interactions between dairy treatment and genotype (**Supplemental Tables 3** and **4**).

No detectable effect of *APOE* or *eNOS* genotype or genotype × treatment interactions were observed for the ∆ postprandial summary measures of lipids (TGs, apoB, and NEFAs), glucose, and insulin (**Supplemental Tables 5** and **6**) or the ∆ AUC or iAUC for circulating markers of endothelial activation (nitrite, nitrate, sVCAM-1, sICAM-1, E-selectin, and P-selectin) and inflammation (TNF-α, IL-6, IL-1β, IL-8, and IL-10) (Supplemental Tables 3 and 4). There were no significant effects of *APOE* or *eNOS* genotype or genotype × treatment interactions for the ∆ AUC or iAUC for any of the plasma total lipid FA responses, including total SFAs, palmitic acid, total *cis*-18:1, oleic acid, total *cis*-MUFAs, total *trans*-18:1, *trans*-MUFAs, and TFAs (data not shown).

## Discussion

Daily consumption of FA-modified dairy products for 12 wk had a beneficial impact on the ∆ iAUC (but not AUC) for the postprandial apoB response but attenuated the %FMD response to sequential FA-modified dairy-rich meals among adults at moderate CVD risk, relative to conventional dairy, independent of *APOE* or *eNOS* genotype.

Endothelial dysfunction, characterized by impaired vascular NO bioavailability, is recognized as a modifiable step in the development and progression of atherosclerosis (7). We previously reported that daily intake of FA-modified dairy products for 12 wk improved the fasted %FMD response and plasma nitrite concentrations (5) and led to substantial incorporation of oleic acid into the plasma phospholipid FA pool among adults with moderate CVD risk (15). Despite a similar improvement in fasted %FMD response to the FA-modified dairy diet within the current report of the same “at risk” cohort (5, 15), we found that the FA-modified dairy diet and representative meals (treatment) attenuated the increase in the iAUC for the postprandial %FMD response and tended to decrease the iAUC for the plasma nitrite response, compared with conventional dairy and relative to preintervention. It is possible that the observed impact of the treatment on ∆ iAUC %FMD response could be linked to the iAUC for plasma SFAs and *cis*-MUFAs not reflecting the FA composition of the specific dairy meals. Indeed, we previously reported that acute exposure to sequential FA-modified, dairy-rich meals led to a postprandial abundance of plasma total lipids that largely reflected the partial replacement of SFAs with MUFAs in the meals, alongside a tendency for a higher AUC for the %FMD response, relative to conventional dairy meals (8). This highlights that altering the background diet (via habituation to diets varying in FA composition) may lead to differential postprandial responses, when compared with manipulating dietary FA intake in an acute manner (9, 33).

It should also be taken into consideration that the attenuated %FMD response following the FA-modified treatment may be linked to the TFA composition of these dairy foods. Our daily FA-modified dietary intervention and 2-meal challenge each naturally contained a 3-g (2-fold) higher *trans*-MUFA content, compared with the conventional treatment (15). In addition, feeding our dairy cows unprotected HOS led to alterations in the *trans*-18:1 isomer profile of our FA-modified dairy products, causing a shift from vaccenic acid, the main *trans* isomer present in conventional milk fat, toward a greater proportion of *trans*-18:1 intermediates, specifically elaidic acid and octadecenoic acid (19). This was reflected in the postprandial plasma lipid pool, with a 36% increase in the ∆ iAUC for elaidic acid following the FA-modified treatment, relative to a 150% decrease following the conventional dairy treatment. Although dairy products are not the most common source of *trans*-9 18:1 intake, increased consumption of elaidic acid (the major *trans* isomer found in partially hydrogenated vegetable oils) is known to adversely affect CVD risk (34). Exposure of human endothelial cells to physiologically relevant concentrations (≤0.1 mM) of elaidic acid for 180 min was linked to increased NF-κB activation and reduced endothelial insulin signaling and NO production, whereas *trans*-vaccenic acid showed no such response (28). This could provide a probable mechanistic explanation for the observed attenuation in the ∆ iAUC for the %FMD response to FA-modified dairy treatment. Our observation is of significance given that repeated fed-state transient endothelial function impairment can adversely affect the atherosclerotic disease process and long-term cardiometabolic health (35, 36). Furthermore, if partial replacement of SFAs with MUFAs in dairy foods is linked to potentially adverse effects, in addition to beneficial impact on the fasting %FMD response (5), the net change in cardiometabolic health outcomes associated with this reformulation initiative would need careful attention before being considered as a public health strategy for CVD risk reduction. However, while meta-analyses of prospective studies have shown that fasting brachial FMD is inversely associated with future CVD events (37, 38), the prognostic value of postprandial FMD for risk of cardiovascular events is less clear.

We found that partial replacement of dietary SFAs with unsaturated FAs in dairy products may have a favorable effect on cardiometabolic disease risk by reducing the postprandial apoB response (a marker of TG-rich lipoproteins). An in vitro competitive cell study demonstrated that enrichment of TG-rich lipoprotein particles with TGs and apoE after an SFA-rich meal led to a reduced uptake of LDL by HepG2 cells as a result of greater competition for LDL receptor mediate uptake, compared with particles isolated after a MUFA-rich meal (39). Observations from this in vitro study (39) could provide a potential mechanism to explain higher circulating LDL cholesterol observed following chronic consumption of dietary SFAs. Thus, it is possible that the lowered ∆ iAUC for the apoB response that we have reported here could explain, in part, the attenuation in fasting LDL-cholesterol concentration that we observed in the same “at risk” cohort following 12-wk FA-modified dairy intake (5). Our finding builds upon previous human studies that suggest beneficial impacts on the kinetics of postprandial lipid responses following 8-wk MUFA-, relative to SFA-, enriched diets (40, 41).

This study had several strengths, including the acute-within-chronic design that allowed us to assess postprandial responses to longer-term ingestion of FA-modified dairy products. The use of a sequential mixed-nutrient meal protocol was also more reflective of real-life eating patterns (31). Reformulation initiatives for partial replacement of SFAs with unsaturated FAs in dairy foods could play an important role in facilitating a transition toward a more sustainable food system (42), particularly given that bovine lipid supplementation strategies may help to mitigate methane emissions from ruminants [for review, see (43)]. Although our ethnic split was largely representative of the UK population, the generalizability of the findings may be limited considering the enrollment of predominantly White individuals at a moderate CVD risk. Dropout from this 32-wk randomized controlled trial was substantial (24 out of 76 participants randomized); however, there was an even split of participants who were unable to comply with the diet (*n* = 4 per study arm), which suggests that both dairy treatments were similar in terms of acceptability (15, 20). Finally, as evaluation of the interactions between *APOE* or *eNOS* genotype and dairy treatment on cardiometabolic markers was not powered, relied on retrospective genotyping, and inevitably resulted in uneven group sizes, particularly in the rarer *APOE4*-carrier group, these findings should be regarded as exploratory in nature.

In conclusion, we found that, among adults at moderate CVD risk, high daily intake of SFA-reduced, MUFA-enriched dairy products for 12 wk beneficially impacted the iAUC for the apoB but reduced the %FMD response to sequential meals, relative to conventional dairy intake, irrespective of *APOE* or *eNOS* genotype. Our findings highlight the importance of looking beyond risk-marker assessment in the fasted state when considering interactions between dietary SFA replacement and cardiometabolic disease risk. A possible reason for the unexpected, potentially adverse, effect of the FA-modified treatment on the postprandial %FMD response may be linked to the high *trans*-MUFA content of these dairy foods. However, further research is necessary to understand the effects of partial replacement of dietary SFAs with *cis*-MUFAs in dairy foods, particularly with those that have been modified to minimize the extent of ruminal formation of elaidic acid (1).

## Supplementary Material

nqab428_Supplemental_File

## Data Availability

Data described in the manuscript will be made available upon reasonable request.

## Notes

Supported by the UK Medical Research Council (MR/K020218/1). Arla Foods UK and AAK UK supplied, in kind, the commercially available dairy products and high-oleic sunflower oil, respectively, according to our specification. The funders of the study had no role in study design; collection, analysis, and interpretation of data; or writing of the manuscript. The supporting source had no such involvement or restrictions regarding publication.

Supplemental Figure 1 and Supplemental Tables 1–6 are available from the “Supplementary data” link in the online posting of the article and from the same link in the online table of contents at https://academic.oup.com/ajcn/.

Present address for OM: School of Sport, Exercise, and Health Sciences, Loughborough University, Loughborough, United Kingdom.

OM and DV contributed equally to this study.

Abbreviations used: CLA, conjugated linoleic acid; Cmax, maximum concentration; Cmin, minimum concentration; CVD, cardiovascular disease; eNOS, endothelial nitric oxide synthase; FA, fatty acid; FAME, fatty acid methyl ester; FMD, flow-mediated dilatation; HOS, high-oleic sunflower oil; iAUC, incremental AUC; IPAQ, International Physical Activity Questionnaire; NEFA, nonesterified fatty acid; RESET, REplacement of SaturatEd fat in dairy on Total cholesterol; sICAM-1, soluble intercellular adhesion molecule-1; sVCAM-1, soluble vascular cell adhesion molecule-1; TC, total cholesterol; TFA, *trans* fatty acid; TG, triacylglycerol; Tmax, time to reach maximum concentration; Tmin, time to reach minimum concentration; UHT, ultra-high temperature; %FMD, percentage of flow-mediated dilatation; %TE, percentage of total energy; *∆*, change from preintervention.
