# Supplementary material for: Effect of fat-reformulated dairy food consumption on postprandial flow-mediated dilatation and cardiometabolic risk biomarkers compared with conventional dairy: a randomized controlled trial
Source: Am J Clin Nutr. 2022 Jan 10;115(3):679–93. doi: 10.1093/ajcn/nqab428 (PMC8895219; doi:10.1093/ajcn/nqab428)
Supplement: nqab428_Supplemental_File [file nqab428_supplemental_file.docx]

**Effect of fat reformulated dairy food consumption on postprandial flow-mediated dilatation and cardiometabolic risk biomarkers compared with conventional dairy: a randomized, controlled trial, Markey et al.**

**Online Supplementary Material**

**Supplemental Table 1** Baseline characteristics of the *APOE* genotype groups combined and according to *E3/E3* and *E4* carrier group^1^

|  | Overall group  (*n* = 44) | *E3/E3* carriers  (*n* = 29) | *E4* carriers  (*n* = 15)^2^ |
| --- | --- | --- | --- |
| Sex (M/F), *n*/*n* | 24/20 | 17/12 | 7/8 |
| Age, y | 52 ± 2 | 50 ± 3 | 56 ± 3 |
| Body mass, kg | 76.8 ± 2.1 | 77.5 ± 2.7 | 75.3 ± 3.3 |
| BMI, kg/m² | 26.0 ± 0.5 | 26.4 ± 0.6 | 25.4 ± 0.9 |
| Waist circumference, cm | 90.0 ± 1.6 | 90.0 ± 2.0 | 90.1 ± 3.0 |
| SBP, mm Hg | 120 ± 2 | 119 ± 3 | 123 ± 2 |
| DBP, mm Hg | 70 ± 1 | 69 ± 1 | 72 ± 2 |
| Fasting serum biomarkers |  |  |  |
| TC, mmol/L | 5.79 ± 0.16 | 5.59 ± 0.17 | 6.16 ± 0.31 |
| LDL-C, mmol/L | 3.52 ± 0.11 | 3.40 ± 0.13 | 3.74 ± 0.18 |
| HDL-C, mmol/L | 1.59 ± 0.05 | 1.51 ± 0.05 | 1.75 ± 0.09 |
| TG, mmol/L | 1.20 ± 0.07 | 1.21 ± 0.08 | 1.18 ± 0.14 |
| Glucose, mmol/L | 5.58 ± 0.12 | 5.54 ± 0.10 | 5.66 ± 0.30 |
| Insulin, pmol/L | 36.0 ± 3.0 | 35.0 ± 3.6 | 38.1 ± 5.5 |
| HOMA-IR | 1.45 ± 0.12 | 1.40 ± 0.16 | 1.53 ± 0.22 |
| CVD risk score^3^ | 3.1 ± 0.2 | 3.1 ± 0.2 | 3.2 ± 0.3 |

^1^Values are unadjusted means ± SEMs or *n* (%). *APOE*, apolipoprotein E; CVD, cardiovascular disease; DBP, diastolic blood pressure; LDL-C, LDL cholesterol; HDL-C, HDL cholesterol; SBP, systolic blood pressure; TG, triacylglycerol; TC, total cholesterol. No significant differences were observed between the genotype groups for any of the baseline characteristics using independent t-tests and chi-square test for continuous and categorical variables, respectively (*P* > 0.01).

^2^*E4* carriers represent: *E3/E4* carriers (*n* = 14) and *E4/E4* carriers (*n* = 1).

^3^Assessed with the use of a modified Framingham risk score, where a score of ≥ 2 points relates to a 50% higher risk of CVD than the population mean (15).

**Supplemental Table 2** Baseline characteristics of the *eNOS* genotype groups combined and according to to Glu298 homozygotes and Asp298

carrier group^1^

|  | Overall group  (*n* = 52) | Glu298 homozygotes  (*n* = 23) | Asp298 carriers  (*n* = 29)^2^ |
| --- | --- | --- | --- |
| Sex (M/F), *n*/*n* | 31/21 | 11/12 | 20/9 |
| Age, y | 53 ± 2 | 52 ± 3 | 54 ± 2 |
| Body mass, kg | 77.5 ± 1.9 | 73.5 ± 3.1 | 80.7 ± 2.2 |
| BMI, kg/m² | 26.0 ± 0.4 | 25.7 ± 0.8 | 26.3 ± 0.5 |
| Waist circumference, cm | 90.6 ± 1.4 | 88.6 ± 2.2 | 92.3 ± 1.9 |
| SBP, mm Hg | 121 ± 2 | 119 ± 3 | 122 ± 2 |
| DBP, mm Hg | 70 ± 1 | 68 ± 2 | 71 ± 1 |
| Fasting serum biomarkers |  |  |  |
| TC, mmol/L | 5.71 ± 0.14 | 5.64 ± 0.23 | 5.77 ± 0.18 |
| LDL-C, mmol/L | 3.48 ± 0.11 | 3.41 ± 0.17 | 3.54 ± 0.14 |
| HDL-C, mmol/L | 1.58 ± 0.04 | 1.62 ± 0.07 | 1.55 ± 0.05 |
| TG, mmol/L | 1.18 ± 0.06 | 1.02 ± 0.08 | 1.30 ± 0.09 |
| Glucose, mmol/L | 5.53 ± 0.11 | 5.34 ± 0.10 | 5.68 ± 0.17 |
| Insulin, pmol/L | 36.4 ± 2.9 | 34.2 ± 4.6 | 38.1 ± 3.6 |
| HOMA-IR | 1.44 ± 0.12 | 1.30 ± 0.18 | 1.55 ± 0.15 |
| CVD risk score^3^ | 3.0 ± 0.2 | 2.8 ± 0.2 | 3.2 ± 0.3 |

^1^Values are unadjusted means ± SEMs or *n*. CVD, cardiovascular disease; DBP, diastolic blood pressure; *eNOS*, enzyme endothelial NO synthase; LDL-C, LDL cholesterol; HDL-C, HDL cholesterol; SBP, systolic blood pressure; TG, triacylglycerol; TC, total cholesterol. No significant differences between any of the baseline characteristics in the genotype groups were observed using independent t-tests and chi-square test for continuous and categorical variables, respectively (*P* > 0.01).

^2^Asp298 carriers represents: Asp298 carrier heterozygotes (GT; *n* = 24) and Asp298 homozygotes (TT; *n* = 5).

^3^Assessed with the use of a modified Framingham risk score, where a score of ≥ 2 points relates to a 50% higher risk of CVD than the population mean (15).

**Supplemental Table 3** Postprandial summary measures for endothelial function and circulating biomarkers of endothelial activation and inflammatory responses to sequential high-fat mixed-meal challenges representative of the fatty acid-modified and conventional (control) dairy diets consumed pre- (wk-0/ wk-20) and post-intervention (wk-12/ wk-32), and the Δ following each 12-wk dietary intervention in adults with moderate cardiovascular disease risk according to according to *APOE* carrier group ^1^

|  | Modified diet and test meal | | | | | | Control diet and test meal | | | | | |  | | |
| --- | --- | --- | --- | --- | --- | --- | --- | --- | --- | --- | --- | --- | --- | --- | --- |
|  | *E3/E3* carriers | | | *E4* carriers | | | *E3/E3* carriers | | | *E4* carriers | | | *P*^2^ | | |
|  | Pre | Post | ∆ | Pre | Post | ∆ | Pre | Post | ∆ | Pre | Post | ∆ | G | T | G x T |
| % FMD response, % x min |  |  |  |  |  |  |  |  |  |  |  |  |  |  |  |
| AUC^3^ | 2100 ± 134 | 2146 ± 129 | 50 ± 86 | 1848 ± 255 | 1824 ± 263 | -24 ± 113 | 2138 ± 143 | 2078 ± 160 | -26 ± 89 | 1553 ± 199 | 1741 ± 281 | 165 ± 145 | 0.850 | 0.843 | 0.385 |
| iAUC | 69 ± 112 | -65 ± 91 | -140 ± 76 | 216 ± 143 | 40 ± 147 | -176 ± 13 | -8 ± 107 | 193 ± 113 | 205 ± 93 | -39 ± 135 | 180 ± 103 | 180 ± 167 | 0.983 | 0.009 | 0.778 |
| Plasma nitrite, μmol/L x min |  |  |  |  |  |  |  |  |  |  |  |  |  |  |  |
| AUC^3^ | 65.0 ± 12.4 | 57.9 ± 12.4 | -7.0 ± 8.5 | 60.3 ± 10.3 | 71.1 ± 13.1 | 10.8 ± 7.8 | 59.6 ± 12.2 | 56.4 ± 16.7 | -0.1 ± 11.1 | 82.3 ± 16.2 | 75.3 ± 16.4 | -7.0 ± 15.9 | 0.188 | 0.816 | 0.465 |
| iAUC^3^ | 3.1 ± 8.2 | -3.6 ± 3.8 | -6.7 ± 8.4 | 6.8 ± 5.4 | -0.1 ± 3.7 | -6.9 ± 4.7 | -6.0 ± 5.1 | 10.4 ± 8.2 | 16.2 ± 9.8 | 8.4 ± 11.2 | 8.7 ± 8.0 | 0.3 ± 13.2 | 0.677 | 0.105 | 0.541 |
| Plasma nitrate, μmol/L x min |  |  |  |  |  |  |  |  |  |  |  |  |  |  |  |
| AUC^3^ | 6380 ± 898 | 5723 ± 504 | -657 ± 642 | 4939 ± 444 | 5546 ± 508 | 607 ± 525 | 5362 ± 392 | 5897 ± 712 | 734 ± 801 | 5416 ± 441 | 5873 ± 730 | 457 ± 538 | 0.375 | 0.800 | 0.814 |
| iAUC^3^ | -1822 ± 418 | -1518 ± 307 | 304 ± 392 | -1625 ± 648 | -2487 ± 962 | -862 ± 1002 | -1593 ± 382 | -1149 ± 394 | 385 ± 493 | -1787 ± 456 | -1283 ± 744 | 504 ± 863 | 0.197 | 0.096 | 0.535 |
| Adhesion molecules, ng/mL x min |  |  |  |  |  |  |  |  |  |  |  |  |  |  |  |
| Plasma sVCAM-1 |  |  |  |  |  |  |  |  |  |  |  |  |  |  |  |
| AUC^3^ | 233 ± 19 | 229 ± 18 | -4 ± 7 | 210 ± 22 | 211 ± 24 | 2 ± 10 | 238 ± 17 | 217 ± 21 | -22 ± 12 | 236 ± 29 | 210 ± 24 | -26 ± 13 | 0.711 | 0.063 | 0.674 |
| iAUC^3^ | -0.8 ± 4.4 | 0.4 ± 6.7 | 1.2 ± 9.3 | 4.9 ± 6.8 | -7.2 ± 7.5 | -12.1 ± 11.2 | 4.6 ± 4.7 | 6.4 ± 3.6 | 1.8 ± 6.8 | 4.3 ± 12.7 | 0.0 ± 4.4 | -4.3 ± 11.5 | 0.907 | 0.424 | 0.243 |
| Plasma sICAM-1 |  |  |  |  |  |  |  |  |  |  |  |  |  |  |  |
| AUC^3^ | 36.3 ± 4.1 | 34.2 ± 3.4 | -2.1 ± 1.7 | 39.3 ± 5.9 | 35.9 ± 5.6 | -3.4 ± 2.1 | 32.1 ± 3.5 | 35.7 ± 4.1 | 3.6 ± 1.5 | 39.2 ± 6.6 | 37.7 ± 7.0 | -1.5 ± 2.3 | 0.055 | 0.397 | 0.379 |
| iAUC^3^ | 2.0 ± 1.4 | -2.6 ± 1.5 | -4.6 ± 2.2 | 2.3 ± 2.3 | 4.9 ± 2.1 | 2.6 ± 10.4 | 1.4 ± 1.6 | -0.6 ± 2.2 | -2.0 ± 3.1 | 0.6 ± 3.7 | -0.3 ± 1.9 | -0.9 ± 3.9 | 0.214 | 0.245 | 0.074 |
| Plasma E-selectin |  |  |  |  |  |  |  |  |  |  |  |  |  |  |  |
| AUC^3^ | 10.7 ± 1.3 | 10.8 ± 1.2 | 0.1 ± 0.3 | 10.0 ± 1.3 | 10.2 ± 1.2 | 0.2 ± 0.3 | 10.4 ± 1.2 | 10.6 ± 1.3 | 0.2 ± 0.4 | 10.2 ± 1.2 | 10.3 ± 1.3 | 0.1 ± 0.4 | 0.801 | 0.852 | 0.136 |
| iAUC^3^ | -0.56 ± 0.18 | -0.39 ± 0.23 | 0.17 ± 0.30 | 0.29 ± 0.36 | -0.22 ± 0.44 | -0.50 ± 0.59 | -0.35 ± 0.13 | -0.21 ± 0.20 | 0.15 ± 0.25 | -0.07 ± 0.42 | -0.31 ± 0.28 | -0.24 ± 0.42 | 0.263 | 0.711 | 0.964 |
| Plasma P-selectin |  |  |  |  |  |  |  |  |  |  |  |  |  |  |  |
| AUC^3^ | 10.9 ± 0.8 | 11.1 ± 0.8 | 0.2 ± 0.2 | 10.3 ± 1.1 | 10.5 ± 1.3 | 0.2 ± 0.5 | 10.6 ± 0.7 | 11.0 ± 0.8 | 0.5 ± 0.3 | 10.5 ± 1.3 | 10.8 ± 1.4 | 0.4 ± 0.5 | 0.987 | 0.604 | 0.974 |
| iAUC^3^ | -0.39 ± 0.17 | -0.31 ± 0.23 | 0.08 ± 0.32 | 0.70 ± 0.36 | -0.77 ± 0.49 | -1.47 ± 0.76 | -0.59 ± 0.22 | -0.06 ± 0.29 | 0.53 ± 0.35 | -0.20 ± 0.43 | 0.12 ± 0.24 | 0.32 ± 0.34 | 0.155 | 0.056 | 0.116 |
| Whole blood culture  LPS-stimulated  cytokines, | |  |  |  |  |  |  |  |  |  |  |  |  |  |  |
| mg x 10^3^ monocytes x min |  |  |  |  |  |  |  |  |  |  |  |  |  |  |  |
| TNF-α |  |  |  |  |  |  |  |  |  |  |  |  |  |  |  |
| AUC^3^ | 5.3 ± 0.5 | 5.8 ± 0.4 | 0.5 ± 0.3 | 4.8 ± 0.6 | 5.3 ± 0.6 | 0.4 ± 0.4 | 5.2 ± 0.4 | 5.8 ± 0.4 | 0.5 ± 0.5 | 4.7 ± 0.5 | 5.2 ± 0.5 | 0.5 ± 0.5 | 0.262 | 0.589 | 0.818 |
| iAUC | -0.1 ± 0.4 | -0.1 ± 0.3 | 0.0 ± 0.5 | -0.3 ± 0.2 | -0.2 ± 0.4 | 0.2 ± 0.5 | 0.2 ± 0.41 | -0.2 ± 0.3 | -0.4 ± 0.4 | 0.1 ± 0.3 | -0.3 ± 0.2 | -0.4 ± 0.4 | 0.916 | 0.844 | 0.981 |
| IL-6 |  |  |  |  |  |  |  |  |  |  |  |  |  |  |  |
| AUC^3^ | 33.9 ± 2.6 | 37.8 ± 2.9 | 3.9 ± 1.5 | 32.7 ± 2.4 | 34.5 ± 3.4 | 1.8 ± 1.9 | 35.0 ± 2.3 | 36.7 ± 2.3 | 1.7 ± 2.2 | 31.3 ± 2.7 | 33.4 ± 3.2 | 2.1 ± 2.6 | 0.291 | 0.313 | 0.612 |
| iAUC | -1.6 ± 1.7 | -2.7 ± 1.7 | -1.1 ± 2.4 | -3.6 ± 1.3 | -0.8 ± 1.9 | 2.8 ± 2.9 | 0.9 ± 2.1 | -1.9 ± 1.2 | -2.8 ± 2.3 | -0.1 ± 2.0 | -0.32 ± 1.5 | -3.1 ± 2.4 | 0.690 | 0.931 | 0.409 |
| IL-1β |  |  |  |  |  |  |  |  |  |  |  |  |  |  |  |
| AUC^3^ | 11.7 ± 0.8 | 12.1 ± 0.9 | 0.5 ± 0.6 | 0.6 ± 0.5 | 0.6 ± 0.5 | -0.1 ± 0.7 | 12.2 ± 0.8 | 12.7 ± 0.6 | 0.5 ± 0.7 | 1.8 ± 0.5 | 1.3 ± 0.6 | -0.6 ± 0.8 | 0.981 | 0.149 | 0.556 |
| iAUC | 13.0 ± 1.0 | 12.5 ± 0.9 | -0.4 ± 0.6 | -0.1 ± 0.7 | 0.1 ± 0.7 | 0.1 ± 1.0 | 12.9 ± 1.3 | 15.5 ± 1.6 | 2.7 ± 1.6 | 0.4 ± 0.5 | 1.2 ± 0.8 | 0.8 ± 1.0 | 0.600 | 0.035 | 0.911 |
| IL-8 |  |  |  |  |  |  |  |  |  |  |  |  |  |  |  |
| AUC | 38.3 ± 5.4 | 41.1 ± 6.9 | 2.8 ± 6.7 | 58.4 ± 6.7 | 63.8 ± 11.6 | 5.4 ± 8.6 | 48.5 ± 9.0 | 42.2 ± 6.3 | -6.3 ± 6.5 | 54.7 ± 9.4 | 60.3 ± 14.3 | 5.6 ± 12.1 | 0.391 | 0.226 | 0.807 |
| iAUC | -5.0 ± 2.6 | -6.4 ± 3.6 | -1.4 ± 4.0 | -2.9 ± 6.7 | -2.4 ± 3.3 | 0.6 ± 6.4 | 1.6 ± 7.0 | 5.5 ± 3.2 | -7.1 ± 4.9 | -10.2 ± 2.2 | -4.3 ± 6.2 | 5.9 ± 6.1 | 0.807 | 0.729 | 0.718 |
| IL-10 |  |  |  |  |  |  |  |  |  |  |  |  |  |  |  |
| AUC | 0.28 ± 0.03 | 0.29 ± 0.04 | 0.01 ± 0.04 | 0.35 ± 0.05 | 0.33 ± 0.05 | -0.02 ± 0.03 | 0.36 ± 0.05 | 0.32 ± 0.04 | -0.04 ± 0.04 | 0.37 ± 0.07 | 0.31 ± 0.06 | -0.05 ± 0.08 | 0.541 | 0.145 | 0.359 |
| iAUC | -0.04 ± 0.02 | -0.05 ± 0.04 | -0.01 ± 0.04 | -0.10 ± 0.05 | -0.11 ± 0.05 | -0.01 ± 0.08 | -0.01 ± 0.03 | -0.07 ± 0.01 | -0.06 ± 0.03 | -0.08 ± 0.02 | -0.12 ± 0.06 | -0.04 ± 0.06 | 0.569 | 0.799 | 0.559 |

^1^Values are untransformed and unadjusted means ± SEMs. For all variables, *n* = 42: *E3/E3* carriers (*n* = 27); *E4* carrier groups were combined for the data analysis: *E3/E4* (*n* = 14) and E4/E4 (*n* = 1); except %FMD response, *n* = 40 (*E3/E3*: *n* = 27; *E3/E4*: *n* = 13), TNF-α, IL-1β, IL-6, IL-10, *n* = 41 (*E3/E3*: *n* = 26; *E3/E4*: *n* = 14; *E4/E4*: *n* = 1) and IL-8, *n* = 39 (*E3/E3*: *n* = 26; *E3/E4*: *n* = 12; *E4/E4*: *n* = 1). The time interval for AUC and iAUC: 420 min for all variables. *APOE*, apolipoprotein E; AUC, area under the curve; G, genotype; G x T, genotype x treatment; iAUC, incremental AUC; sICAM-1, soluble intercellular adhesion molecule-1; T, treatment; TNF-α, tumor necrosis factor alpha; sVCAM-1, soluble vascular adhesion molecule-1; %FMD, percentage of flow-mediated dilatation; Δ, change-from-pre-intervention.

^2^Linear mixed-model analyses were used to calculate overall treatment effect based on Δ in each 12-wk dietary intervention (calculated by subtracting week 0 from week 12 values; and week 20 from week 32 values), with adjustments made for fixed effects of baseline values of the assessed outcome measure at the beginning of each dietary period (i.e., the postprandial summary measure for the pre-intervention visit), period, treatment, sex, genotype, age, BMI, and genotype x treatment. Participant was included as a random effect. No period effects were observed in the model for any outcome measure. *P* < 0.05 was deemed significant for %FMD response (primary outcome). For all outcome measures for the secondary variables, *P* ≤ 0.01 was deemed significant to acknowledge multiplicity.

^3^Indicates data were log transformed prior to analysis.

**Supplemental Table 4** Postprandial summary measures for endothelial function and circulating biomarkers of endothelial activation and inflammatory responses to sequential high-fat mixed-meal challenges representative of the fatty acid-modified and conventional (control) dairy diets consumed pre- (wk-0/ wk-20) and post-intervention (wk-12/ wk-32), and the Δ following each 12-wk dietary intervention in adults with moderate cardiovascular disease risk according to Glu298 homozygotes and Asp298 carrier group^1^

|  | Modified diet and test meal | | | | | | Control diet and test meal | | | | | |  |  |  |
| --- | --- | --- | --- | --- | --- | --- | --- | --- | --- | --- | --- | --- | --- | --- | --- |
|  | Glu298 homozygotes | | | Asp298 carriers | | | Glu298 homozygotes | | | Asp298 carriers | | | *P*^2^ | | |
|  | Pre | Post | ∆ | Pre | Post | ∆ | Pre | Post | ∆ | Pre | Post | ∆ | G | T | G x T |
| % FMD response, % x min |  |  |  |  |  |  |  |  |  |  |  |  |  |  |  |
| AUC^3^ | 1920 ± 153 | 1944 ± 161 | 24 ± 92 | 2102 ± 150 | 2075 ± 141 | -26 ± 83 | 1918 ± 142 | 1902 ± 182 | -12 ± 106 | 1990 ± 160 | 1996 ± 164 | 6 ± 26 | 0.667 | 0.65 | 0.568 |
| iAUC | 199 ± 110 | 5 ± 107 | -194 ± 86 | 81 ± 108 | 1 ± 101 | -85 ± 106 | 103 ± 127 | 205 ± 138 | 76 ± 110 | -80 ± 101 | 209 ± 95 | 289 ± 115 | 0.404 | 0.007 | 0.719 |
| Plasma nitrite, μmol/L x min |  |  |  |  |  |  |  |  |  |  |  |  |  |  |  |
| AUC^3^ | 60.0 ± 9.0 | 69.0 ± 10.6 | 9.0 ± 4.3 | 66.9 ± 12.1 | 61.0 ± 12.2 | -5.9 ± 8.7 | 68.2 ± 12.2 | 59.0 ± 11.0 | -9.2 ± 11.2 | 63.3 ± 12.1 | 60.5 ± 16.6 | -0.6 ± 11.7 | 0.254 | 0.459 | 0.311 |
| iAUC^3^ | -3.3 ± 5.1 | -2.7 ± 5.1 | 0.6 ± 6.4 | 10.1 ± 7.4 | -1.8 ± 2.3 | -11.9 ± 7.3 | 3.9 ± 8.9 | 6.5 ± 4.0 | 2.7 ± 9.3 | -4.7 ± 4.3 | 12.9 ± 8.7 | 17.4 ± 9.7 | 0.378 | 0.028 | 0.887 |
| Plasma nitrate, μmol/L x min |  |  |  |  |  |  |  |  |  |  |  |  |  |  |  |
| AUC^3^ | 4989 ± 397 | 5635 ± 455 | 646 ± 379 | 6285 ± 861 | 5714 ± 474 | -572 ± 638 | 5121 ± 399 | 5409 ± 623 | 289 ± 541 | 5424 ± 374 | 5877 ± 669 | -572 ± 638 | 0.385 | 0.314 | 0.476 |
| iAUC^3^ | -2035 ± 373 | -1870 ± 689 | 165 ± 726 | -1358 ± 452 | -1627 ± 283 | -269 ± 375 | -1650 ± 350 | -1416 ± 513 | 234 ± 496 | -1747 ± 354 | -958 ± 385 | 726 ± 538 | 0.568 | 0.106 | 0.543 |
| Adhesion molecules, ng/mL x min |  |  |  |  |  |  |  |  |  |  |  |  |  |  |  |
| Plasma sVCAM-1 |  |  |  |  |  |  |  |  |  |  |  |  |  |  |  |
| AUC^3^ | 213 ± 16 | 216 ± 17 | 3 ± 6 | -2.5 ± 4.9 | 2.1 ± 6.3 | 4.5 ± 8.9 | 222 ± 18 | 196 ± 19 | -25 ± 14 | -2.1 ± 6.4 | 3.8 ± 4.6 | 5.9 ± 7.6 | 0.434 | 0.151 | 0.867 |
| iAUC^3^ | 236 ± 18 | 231 ± 17 | -5 ± 8 | 0.5 ± 4.7 | -6.3 ± 6.1 | -6.8 ± 9.1 | 248 ± 19 | 235 ± 20 | -14 ± 9 | 8.3 ± 6.3 | 2.7 ± 2.7 | -5.6 ± 6.8 | 0.658 | 0.425 | 0.071 |
| Plasma sICAM-1 |  |  |  |  |  |  |  |  |  |  |  |  |  |  |  |
| AUC^3^ | 40.6 ± 4.6 | 40.1 ± 4.7 | -0.5 ± 1.9 | 35.5 ± 3.9 | 32.3 ± 3.0 | -3.2 ± 1.5 | 37.3 ± 4.8 | 42.6 ± 5.3 | 5.2 ± 1.7 | 35.6 ± 4.1 | 33.7 ± 4.1 | -2.0 ± 1.8 | 0.303 | 0.409 | 0.943 |
| iAUC^3^ | 0.7 ± 2.2 | 3.7 ± 2.5 | 3.0 ± 3.6 | 0.7 ± 1.3 | -1.2 ± 1.4 | -1.8 ± 2.2 | -0.9 ± 2.9 | 1.1 ± 2.1 | 1.9 ± 3.4 | 2.5 ± 1.4 | -2.4 ± 1.8 | -4.9 ± 2.9 | 0.552 | 0.245 | 0.203 |
| Plasma E-selectin |  |  |  |  |  |  |  |  |  |  |  |  |  |  |  |
| AUC^3^ | 9.1 ± 0.9 | 9.4 ± 1.0 | 0.3 ± 0.3 | 11.3 ± 1.3 | 11.3 ± 1.1 | 0.0 ± 0.3 | 9.1 ± 0.9 | 9.3 ± 1.1 | 0.2 ± 0.4 | 11.2 ± 1.1 | 11.4 ± 1.2 | 0.2 ± 0.3 | 0.961 | 0.758 | 0.482 |
| iAUC^3^ | -0.51 ± 0.22 | -0.43 ± 0.18 | 0.08 ± 0.25 | -0.11 ± 0.23 | -0.23 ± 0.29 | -0.12 ± 0.40 | -0.49 ± 0.15 | -0.01 ± 0.14 | 0.48 ± 0.23 | -0.10 ± 0.24 | -0.32 ± 0.23 | -0.22 ± 0.30 | 0.806 | 0.496 | 0.229 |
| Plasma P-selectin |  |  |  |  |  |  |  |  |  |  |  |  |  |  |  |
| AUC^3^ | 11.5 ± 1.0 | 12.0 ± 1.1 | 0.5 ± 0.3 | 10.1 ± 0.7 | 10.1 ± 0.7 | 0.0 ± 0.2 | 11.6 ± 1.0 | 12.1 ± 1.2 | 0.5 ± 0.4 | 10.0 ± 0.7 | 10.3 ± 0.8 | 0.3 ± 0.3 | 0.929 | 0.876 | 0.808 |
| iAUC^3^ | -0.08 ± 0.31 | -0.30 ± 0.34 | -0.21 ± 0.55 | -0.02 ± 0.17 | -0.50 ± 0.24 | -0.48 ± 0.33 | -0.65 ± 0.28 | 0.49 ± 0.38 | 1.14 ± 0.42 | -0.40 ± 0.23 | -0.28 ± 0.16 | 0.12 ± 0.29 | 0.490 | 0.327 | 0.771 |
| Whole blood culture LPS-stimulated cytokines,  mg x 103 monocytes x min | |  |  |  |  |  |  |  |  |  |  |  |  |  |  |
|  |  |  |  |  |  |  |  |  |  |  |  |  |  |  |  |
| TNF-α |  |  |  |  |  |  |  |  |  |  |  |  |  |  |  |
| AUC^3^ | 5.4 ± 0.5 | 6.0 ± 0.5 | 0.6 ± 0.3 | 4.6 ± 0.4 | 4.9 ± 0.3 | 0.4 ± 0.3 | 5.1 ± 0.4 | 5.9 ± 0.5 | 0.8 ± 0.5 | 4.9 ± 0.4 | 5.2 ± 0.4 | 0.4 ± 0.5 | 0.375 | 0.876 | 0.880 |
| iAUC | -0.21 ± 0.38 | -0.35 ± 0.26 | -0.14 ± 0.34 | -0.14 ± 0.21 | -0.05 ± 0.32 | 0.09 ± 0.43 | -0.16 ± 0.24 | -0.33 ± 0.26 | -0.17 ± 0.36 | 0.43 ± 0.38 | -0.20 ± 0.22 | -0.61 ± 0.37 | 0.938 | 0.919 | 0.482 |
| IL-6 |  |  |  |  |  |  |  |  |  |  |  |  |  |  |  |
| AUC^3^ | 36.2 ± 3.1 | 39.5 ± 3.5 | 3.3 ± 1.3 | 30.0 ± 1.6 | 32.1 ± 2.1 | 2.1 ± 1.5 | 35.8 ± 2.8 | 38.3 ± 2.8 | 2.6 ± 2.4 | 30.8 ± 2.0 | 32.2 ± 2.1 | 1.4 ± 2.2 | 0.652 | 0.971 | 0.759 |
| iAUC | -1.80 ± 1.78 | -3.11 ± 0.94 | -1.31 ± 1.81 | -2.43 ± 1.06 | -1.35 ± 1.80 | 1.08 ± 2.46 | -0.30 ± 1.44 | -3.53 ± 1.45 | -3.23 ± 2.16 | 1.10 ± 2.09 | -1.95 ± 1.05 | -3.05 ± 2.06 | 0.584 | 0.481 | 0.968 |
| IL-1β |  |  |  |  |  |  |  |  |  |  |  |  |  |  |  |
| AUC^3^ | 12.8 ± 0.9 | 13.2 ± 0.9 | 0.4 ± 0.6 | 11.1 ± 0.7 | 11.0 ± 0.7 | -0.1 ± 0.5 | 13.1 ± 0.9 | 14.0 ± 0.9 | 0.9 ± 0.9 | 12.0 ± 0.8 | 13.1 ± 1.0 | 1.5 ± 0.9 | 0.598 | 0.156 | 0.700 |
| iAUC | 0.26 ± 0.55 | 0.78 ± 0.51 | 0.51 ± 0.79 | 0.38 ± 0.46 | 0.32 ± 0.45 | -0.06 ± 0.64 | 2.22 ± 0.56 | 1.07 ± 0.72 | -1.15 ± 0.95 | 0.90 ± 0.45 | 1.38 ± 0.46 | 0.52 ± 0.67 | 0.657 | 0.067 | 0.595 |
| IL-8 |  |  |  |  |  |  |  |  |  |  |  |  |  |  |  |
| AUC | 49.6 ± 7.0 | 54.9 ± 9.5 | 5.0 ± 5.1 | 48.2 ± 4.5 | 53.5 ± 7.0 | 3.0 ± 7.1 | 46.5 ± 6.1 | 49.0 ± 10.2 | 0.1 ± 7.8 | 55.3 ± 9.5 | 48.9 ± 6.2 | -2.1 ± 6.4 | 0.490 | 0.161 | 0.466 |
| iAUC | -3.10 ± 3.33 | -5.94 ± 4.45 | -2.71 ± 5.03 | -5.30 ± 3.56 | -0.38 ± 4.72 | 4.74 ± 4.46 | -3.37 ± 2.86 | -9.96 ± 3.68 | -6.11 ± 3.99 | -3.42 ± 7.49 | -4.58 ± 3.72 | -1.43 ± 5.19 | 0.196 | 0.171 | 0.484 |
| IL-10 |  |  |  |  |  |  |  |  |  |  |  |  |  |  |  |
| AUC | 0.27 ± 0.03 | 0.26 ± 0.03 | -0.01 ± 0.04 | 0.31 ± 0.04 | 0.33 ± 0.04 | 0.03 ± 0.03 | 0.28 ± 0.04 | 0.27 ± 0.04 | -0.01 ± 0.05 | 0.42 ± 0.05 | 0.35 ± 0.04 | -0.05 ± 0.05 | 0.234 | 0.281 | 0.664 |
| iAUC | -0.03 ± 0.03 | -0.07 ± 0.03 | -0.04 ± 0.04 | -0.08 ± 0.03 | -0.06 ± 0.04 | 0.02 ± 0.05 | -0.03 ± 0.01 | -0.07 ± 0.02 | -0.04 ± 0.02 | -0.03 ± 0.03 | -0.10 ± 0.03 | -0.07 ± 0.04 | 0.791 | 0.913 | 0.548 |

^1^Values are untransformed and unadjusted means ± SEMs. For all variables, *n* = 50: Glu298 homozygotes (GG: *n* = 22); Asp298 carriers represents: Asp298 heterozygotes (GT; *n* = 23) and Asp298 homozygotes (TT; *n* = 5); except %FMD response, *n* = 47 (GG: *n* = 21; GT: *n* = 21; TT: *n* = 5), TNF-α, IL-1β, IL-6, IL-10, *n* = 49 (GG: *n* = 22; GT: *n* = 23; TT: *n* = 4) and IL-8, *n* = 47 (GG: *n* = 21; GT: *n* = 22; TT: *n* = 4). The time interval for AUC and iAUC: 420 min for all variables. AUC, area under the curve; G, genotype; G x T, genotype x treatment; iAUC, incremental AUC; sICAM-1, soluble intercellular adhesion molecule-1; T, treatment; TNF-α, tumor necrosis factor alpha; sVCAM-1, soluble vascular adhesion molecule-1; %FMD, percentage of flow-mediated dilatation; Δ, change-from-pre-intervention.

^2^Linear mixed-model analyses were used to calculate overall treatment effect based on Δ in each 12-wk dietary intervention (calculated by subtracting week 0 from week 12 values; and week 20 from week 32 values), with adjustments made for fixed effects of baseline values of the assessed outcome measure at the beginning of each dietary period (i.e., the postprandial summary measure for the pre-intervention visit), period, treatment, sex, genotype, age, BMI, and genotype x treatment. Participant was included as a random effect. No period effects were observed in the model for any outcome measure. *P* < 0.05 was deemed significant for %FMD response (primary outcome). For all outcome measures for the secondary variables, *P* ≤ 0.01 was deemed significant to acknowledge multiplicity.

^3^Indicates data were log transformed prior to analysis.

**Supplemental Table 5** Postprandial summary measures for serum lipids, glucose, and insulin responses to sequential high-fat mixed-meal challenges representative of the fatty acid-modified and conventional (control) dairy diets consumed pre- (wk-0/ wk-20) and post-intervention (wk-12/ wk-32), and the Δ following each 12-wk dietary intervention in adults with moderate cardiovascular disease risk according to *APOE* carrier group^1^

|  | Modified diet and test meal | | | | | | Control diet and test meal | | | | | |  |  | |
| --- | --- | --- | --- | --- | --- | --- | --- | --- | --- | --- | --- | --- | --- | --- | --- |
|  | *E3/E3* carriers | | | *E4* carriers | | | *E3/E3* carriers | | | *E4* carriers | | | *P^2^* | | |
|  | Pre | Post | ∆ | Pre | Post | ∆ | Pre | Post | ∆ | Pre | Post | ∆ | G | T | G x T |
| TG |  |  |  |  |  |  |  |  |  |  |  |  |  |  |  |
| Cmax, mmol/L | 2.93 ± 0.20 | 2.90 ± 0.27 | 0.00 ± 0.18 | 2.49 ± 0.21 | 3.01 ± 0.21 | 0.55 ± 0.26 | 2.70 ± 0.15 | 2.66 ± 0.19 | 0.06 ± 0.15 | 2.45 ± 0.25 | 2.32 ± 0.18 | -0.09 ± 0.17 | 0.342 | 0.159 | 0.785 |
| Tmax, min | 357 ± 11 | 338 ± 12 | -18 ± 16 | 353 ± 19 | 315 ± 20 | -28 ± 20 | 342 ± 10 | 332 ± 17 | -7 ± 19 | 324 ± 20 | 309 ± 20 | -16 ± 30 | 0.406 | 0.277 | 0.481 |
| AUC, mmol/L x min^3^ | 992 ± 72 | 1012 ± 105 | 31 ± 69 | 815 ± 62 | 1012 ± 89 | 191 ± 87 | 914 ± 55 | 935 ± 75 | 59 ± 65 | 811 ± 87 | 811 ± 75 | 77 ± 100 | 0.278 | 0.065 | 0.065 |
| iAUC, mmol/L x min^3^ | 363 ± 33 | 390 ± 45 | 27 ± 37 | 305 ± 36 | 388 ± 45 | 84 ± 39 | 341 ± 29 | 351 ± 39 | 20 ± 28 | 282 ± 47 | 294 ± 37 | 14 ± 35 | 0.424 | 0.387 | 0.707 |
| apoB |  |  |  |  |  |  |  |  |  |  |  |  |  |  |  |
| Cmax, mg/mL | 1.03 ± 0.04 | 1.04 ± 0.04 | 0.01 ± 0.02 | 1.05 ± 0.06 | 1.09 ± 0.05 | 0.02 ± 0.03 | 1.02 ± 0.04 | 1.07 ± 0.04 | 0.05 ± 0.02 | 1.03 ± 0.05 | 1.09 ± 0.05 | 0.05 ± 0.03 | 0.360 | 0.712 | 0.489 |
| Tmax, min | 189 ± 28 | 183 ± 28 | -10 ± 41 | 214 ± 41 | 268 ± 38 | 60 ± 45 | 242 ± 27 | 242 ± 30 | 0 ± 38 | 141 ± 28 | 204 ± 43 | 51 ± 47 | 0.393 | 0.985 | 0.207 |
| AUC, mg/mL x min^3^ | 475 ± 18 | 470 ± 19 | -1 ± 10 | 480 ± 23 | 492 ± 22 | 9 ± 11 | 461 ± 20 | 482 ± 20 | 21 ± 7 | 475 ± 20 | 500 ± 21 | 21 ± 11 | 0.638 | 0.091 | 0.687 |
| iAUC, mg/mL x min^3^ | -15 ± 4 | -21 ± 4 | -4 ± 4 | -21 ± 7 | -18 ± 5 | 3 ± 7 | -13 ± 3 | -6 ± 3 | 7 ± 4 | -11 ± 4 | -13 ± 4 | -1 ± 6 | 0.917 | 0.027 | 0.299 |
| NEFAs |  |  |  |  |  |  |  |  |  |  |  |  |  |  |  |
| Cmin_30–330,_ μmol/L^3^ | 110 ± 7 | 108 ± 9 | -4 ± 7 | 109 ± 10 | 104 ± 9 | -7 ± 12 | 104 ± 8 | 105 ± 7 | 3 ± 7 | 112 ± 10 | 127 ± 14 | 19 ± 12 | 0.628 | 0.081 | 0.781 |
| Tmin_30–330,_ min^3^ | 144 ± 8 | 128 ± 7 | -17 ± 10 | 125 ± 10 | 129 ± 10 | 13 ± 12 | 133 ± 8 | 124 ± 7 | -8 ± 11 | 126 ± 17 | 135 ± 15 | 9 ± 9 | 0.148 | 0.693 | 0.677 |
| Suppression_30–330,_ %^3^ | 80 ± 1 | 78 ± 2 | 2 ± 2 | 77 ± 5 | 76 ± 2 | 2 ± 3 | 78 ± 2 | 77 ± 2 | 5 ± 3 | 77 ± 5 | 76 ± 2 | 2 ± 4 | 0.781 | 0.270 | 0.681 |
| Cmax_120-480,_ μmol/L^3^ | 443 ± 28 | 457 ± 29 | 12 ± 33 | 476 ± 35 | 472 ± 41 | -18 ± 36 | 456 ± 24 | 457 ± 25 | -2 ± 30 | 563 ± 44 | 491 ± 26 | -64 ± 49 | 0.965 | 0.594 | 0.573 |
| Tmax_120-480,_ min | 364 ± 7 | 293 ± 25 | -77 ± 28 | 355 ± 6 | 362 ± 5 | 7 ± 8 | 362 ± 6 | 357 ± 6 | -3 ± 8 | 347 ± 15 | 356 ± 5 | 9 ± 16 | 0.111 | 0.247 | 0.046 |
| AUC_120-480,_ mmol/L x min^3^ | 92 ± 5 | 96 ± 6 | 4 ± 5 | 96 ± 8 | 95 ± 7 | -5 ± 5 | 93 ± 4 | 94± 4 | 1 ± 5 | 110 ± 28 | 104 ± 26 | -5 ± 7 | 0.667 | 0.444 | 0.463 |
| iAUC_120-480,_ mmol/L x min^3^ | 48 ± 4 | 52 ± 5 | 6 ± 4 | 49 ± 8 | 52 ± 7 | 1 ± 9 | 53 ± 4 | 52 ± 5 | -1 ± 5 | 62 ± 10 | 55 ± 4 | -7 ± 8 | 0.579 | 0.841 | 0.701 |
| Glucose |  |  |  |  |  |  |  |  |  |  |  |  |  |  |  |
| Cmax, mmol/L^3^ | 8.24 ± 0.25 | 8.19 ± 0.20 | -0.39 ± 0.29 | 8.63 ± 0.46 | 9.13 ± 0.46 | 0.43 ± 0.35 | 7.94 ± 0.25 | 8.16 ± 0.21 | 0.26 ± 0.20 | 8.48 ± 0.47 | 8.38 ± 0.58 | -0.09 ± 0.36 | 0.659 | 0.422 | 0.375 |
| Tmax, min | 229 ± 31 | 270 ± 32 | 37 ± 38 | 222 ± 50 | 257 ± 47 | 28 ± 50 | 263 ± 31 | 274 ± 31 | -4 ± 32 | 208 ± 47 | 304 ± 42 | 63 ± 57 | 0.892 | 0.999 | 0.493 |
| AUC, mmol/L x min^3^ | 2905 ± 55 | 2887 ± 47 | -16 ± 39 | 3083 ± 174 | 3135 ± 169 | 58 ± 68 | 2822 ± 52 | 2885 ± 50 | 92 ± 37 | 3010 ± 163 | 3006 ± 267 | 21 ± 121 | 0.130 | 0.222 | 0.101 |
| iAUC, mmol/L x min | 336 ± 50 | 302 ± 45 | -33 ± 53 | 384 ± 69 | 458 ± 98 | 80 ± 65 | 262 ± 53 | 331 ± 43 | 74 ± 60 | 271 ± 101 | 289 ± 71 | 54 ± 117 | 0.483 | 0.438 | 0.126 |
| Insulin |  |  |  |  |  |  |  |  |  |  |  |  |  |  |  |
| Cmax, pmol/L | 506 ± 46 | 516 ± 41 | 19 ± 22 | 673 ± 138 | 730 ± 157 | 59 ± 61 | 488 ± 35 | 486 ± 32 | -3 ± 28 | 573 ± 100 | 512 ± 56 | -74 ± 69 | 0.508 | 0.118 | 0.137 |
| Tmax, min | 193 ± 31 | 198 ± 30 | -7 ± 40 | 152 ± 45 | 221 ± 46 | 58 ± 51 | 218 ± 29 | 170 ± 28 | -60 ± 25 | 242 ± 42 | 193 ± 44 | -69 ± 72 | 0.592 | 0.275 | 0.685 |
| AUC, µmol/L x min^3^ | 114 ± 10 | 109 ± 8 | -3 ± 5 | 135 ± 29 | 147 ± 39 | 12 ± 16 | 108 ± 8 | 108 ± 7 | 2 ± 3 | 119 ± 20 | 109 ± 14 | -9 ± 7 | 0.916 | 0.810 | 0.542 |
| iAUC, µmol/L x min^3^ | 96 ± 8 | 91 ± 7 | -4 ± 5 | 113 ± 25 | 126 ± 37 | 13 ± 17 | 90 ± 7 | 86 ± 7 | -2 ± 4 | 101 ± 18 | 92 ± 13 | -9 ± 7 | 0.587 | 0.915 | 0.832 |

^1^Values are untransformed and unadjusted means ± SEMs. For all variables, *n* = 44: *E3/E3* carriers (*n* = 29); *E4* carriers represents: *E3/E4* carriers (*n* = 14) and *E4/E4* carriers (*n* = 1). Time interval for AUC and iAUC: 480 min for all variables, except for 360 min NEFA. *APOE*, apolipoprotein E; AUC, area under the curve; Cmax, maximum concentration; Cmin, minimum concentration; G, genotype; G x T, genotype x treatment; iAUC, incremental AUC; NEFA, nonesterified fatty acid; T, treatment; TG; triacylglycerol; Tmax, time to reach maximum concentration; Tmin, time to reach minimum concentration; Δ, change-from-pre-intervention.

^2^Linear mixed-model analyses were used to calculate overall treatment effect based on Δ in each 12-wk dietary intervention (calculated by subtracting week 0 from week 12 values; and week 20 from week 32 values), with adjustments made for fixed effects of baseline values of the assessed outcome measure at the beginning of each dietary period (i.e., the postprandial summary measure for the pre-intervention visit), period, treatment, sex, genotype, age, BMI, and genotype x treatment. Participant was included as a random effect. No period effects were observed in the model for any outcome measure. For all outcome measures, *P* ≤ 0.01 was deemed significant to acknowledge multiplicity.

^3^Indicates data were log transformed prior to analysis.

**Supplemental Table 6** Postprandial measures for serum lipids, glucose, and insulin responses to sequential high-fat mixed-meal challenges representative of the fatty acid-modified and conventional (control) dairy diets consumed pre- (wk-0/ wk-20) and post-intervention (wk-12/ wk-32), and the Δ following each 12-wk dietary intervention in adults with moderate cardiovascular disease risk according to Glu298 homozygotes and Asp298 carrier group^1^

|  | Modified diet and test meal | | | | | | Control diet and test meal | | | | | |  | | |
| --- | --- | --- | --- | --- | --- | --- | --- | --- | --- | --- | --- | --- | --- | --- | --- |
|  | Glu298 homozygotes | | | Asp298 carriers | | | Glu298 homozygotes | | | Asp298 carriers | | | *P*^2^ | | |
|  | Pre | Post | ∆ | Pre | Post | ∆ | Pre | Post | ∆ | Pre | Post | ∆ | G | T | G x T |
| TG |  |  |  |  |  |  |  |  |  |  |  |  |  |  |  |
| Cmax, mmol/L | 2.87 ± 0.21 | 2.60 ± 0.20 | -0.23 ± 0.21 | 2.82 ± 0.21 | 3.16 ± 0.27 | 0.36 ± 0.20 | 2.48 ± 0.19 | 2.56 ± 0.19 | 0.08 ± 0.12 | 2.67 ± 0.16 | 2.57 ± 0.20 | 0.04 ± 0.19 | 0.296 | 0.590 | 0.108 |
| Tmax, min | 360 ± 14 | 332 ± 14 | -28 ± 15 | 358 ± 13 | 329 ± 14 | -23 ± 16 | 329 ± 12 | 335 ± 17 | 7 ± 17 | 338 ± 14 | 314 ± 18 | -20 ± 23 | 0.695 | 0.723 | 0.296 |
| AUC, mmol/L x min^3^ | 933 ± 72 | 883 ± 81 | -35 ± 79 | 969 ± 75 | 1114 ± 107 | 147 ± 74 | 816 ± 57 | 882 ± 74 | 66 ± 43 | 932 ± 61 | 928 ± 80 | 83 ± 81 | 0.516 | 0.317 | 0.183 |
| iAUC, mmol/L x min^3^ | 366 ± 39 | 366 ± 31 | -3 ± 36 | 345 ± 34 | 404 ± 47 | 60 ± 40 | 309 ± 37 | 353 ± 40 | 44 ± 23 | 331 ± 28 | 313 ± 36 | -5 ± 33 | 0.298 | 0.157 | 0.202 |
| apoB |  |  |  |  |  |  |  |  |  |  |  |  |  |  |  |
| Cmax, mg/mL | 1.01 ± 0.05 | 1.03 ± 0.05 | 0.03 ± 0.02 | 1.06 ± 0.04 | 1.07 ± 0.04 | 0.01 ± 0.02 | 1.01 ± 0.05 | 1.03 ± 0.05 | 0.02 ± 0.02 | 1.05 ± 0.04 | 1.10 ± 0.04 | 0.06 ± 0.02 | 0.587 | 0.469 | 0.401 |
| Tmax, min | 240 ± 36 | 204 ± 35 | -44 ± 56 | 185 ± 30 | 211 ± 29 | 26 ± 35 | 230 ± 35 | 210 ± 35 | -20 ± 38 | 193 ± 27 | 236 ± 31 | 38 ± 42 | 0.837 | 0.390 | 0.775 |
| AUC, mg/mL x min^3^ | 462 ± 21 | 463 ± 23 | 6 ± 10 | 485 ± 19 | 487 ± 20 | 2 ± 9 | 453 ± 22 | 471 ± 23 | 18 ± 7 | 479 ± 18 | 500 ± 18 | 19 ± 9 | 0.880 | 0.075 | 0.919 |
| iAUC, mg/mL x min^3^ | -11 ± 4 | -20 ± 4 | -8 ± 4 | -19 ± 5 | -20 ± 4 | -1 ± 5 | -11 ± 3 | -12 ± 3 | -1 ± 4 | -11 ± 3 | -6 ± 4 | 6 ± 5 | 0.257 | 0.005 | 0.508 |
| NEFAs |  |  |  |  |  |  |  |  |  |  |  |  |  |  |  |
| Cmin_30–330,_ μmol/L^3^ | 120 ± 8 | 121 ± 9 | 0 ± 10 | 109 ± 8 | 104 ± 8 | -5 ± 7 | 110 ± 7 | 127 ± 10 | 17 ± 8 | 109 ± 8 | 111 ± 8 | 6 ± 8 | 0.247 | 0.068 | 0.791 |
| Tmin_30–330,_ min^3^ | 149 ± 9 | 142 ± 8 | -14 ± 13 | 132 ± 6 | 120 ± 6 | -8 ± 9 | 142 ± 7 | 130 ± 8 | -12 ± 8 | 126 ± 11 | 131 ± 10 | 4 ± 12 | 0.982 | 0.634 | 0.029 |
| Suppression_30–330,_ %^3^ | 80 ± 2 | 77 ± 1 | 2 ± 2 | 78 ± 2 | 77 ± 2 | 1 ± 2 | 81 ± 1 | 75 ± 2 | 6 ± 2 | 75 ± 3 | 76 ± 2 | 2 ± 4 | 0.218 | 0.117 | 0.108 |
| Cmax_120-480,_ μmol/L^3^ | 420 ± 22 | 449 ± 23 | 29 ± 20 | 458 ± 29 | 455 ± 33 | -11 ± 34 | 476 ± 21 | 442 ± 18 | -34 ± 26 | 489 ± 35 | 473 ± 28 | -15 ± 40 | 0.475 | 0.908 | 0.227 |
| Tmax_120-480,_ min | 365 ± 8 | 319 ± 24 | -53 ± 26 | 358 ± 6 | 324 ± 20 | -36 ± 23 | 353 ± 6 | 356 ± 8 | 3 ± 9 | 362 ± 9 | 352 ± 5 | -9 ± 11 | 0.998 | 0.085 | 0.774 |
| AUC_120-480,_ mmol/L x min^3^ | 90 ± 4 | 95 ± 4 | 6 ± 4 | 94 ± 4 | 95 ± 4 | -1 ± 4 | 93 ± 4 | 95 ± 4 | 1 ± 4 | 100 ± 4 | 99 ± 4 | 0 ± 4 | 0.228 | 0.700 | 0.217 |
| iAUC_120-480,_ mmol/L x min^3^ | 40 ± 4 | 46 ± 5 | 8 ± 3 | 50 ± 4 | 53 ± 5 | 1 ± 3 | 53 ± 4 | 44 ± 3 | -9 ± 4 | 52 ± 4 | 56 ± 3 | 3 ± 4 | 0.592 | 0.475 | 0.740 |
| Glucose |  |  |  |  |  |  |  |  |  |  |  |  |  |  |  |
| Cmax, mmol/L^3^ | 7.84 ± 0.29 | 8.27 ± 0.32 | -0.01 ± 0.46 | 8.58 ± 0.28 | 8.46 ± 0.26 | -0.18 ± 0.18 | 7.77 ± 0.22 | 7.72 ± 0.16 | -0.06 ± 0.22 | 8.24 ± 0.31 | 8.40 ± 0.39 | 0.18 ± 0.22 | 0.451 | 0.159 | 0.257 |
| Tmax, min | 261 ± 35 | 243 ± 37 | -26 ± 43 | 193 ± 32 | 269 ± 33 | 72 ± 33 | 257 ± 35 | 300 ± 33 | 43 ± 37 | 246 ± 33 | 275 ± 34 | -10 ± 34 | 0.806 | 0.690 | 0.039 |
| AUC, mmol/L x min^3^ | 2865 ± 60 | 2870 ± 70 | 12 ± 50 | 2994 ± 97 | 2964 ± 97 | -33 ± 42 | 2786 ± 54 | 2802 ± 48 | 16 ± 50 | 2933 ± 98 | 2967 ± 157 | 71 ± 69 | 0.434 | 0.503 | 0.260 |
| iAUC, mmol/L x min | 381 ± 69 | 451 ± 83 | 75 ± 94 | 317 ± 45 | 291 ± 58 | -29 ± 55 | 323 ± 55 | 350 ± 55 | 27 ± 47 | 211 ± 62 | 259 ± 52 | 79 ± 69 | 0.137 | 0.428 | 0.188 |
| Insulin |  |  |  |  |  |  |  |  |  |  |  |  |  |  |  |
| Cmax, pmol/L | 532 ± 90 | 588 ± 120 | 68 ± 46 | 581 ± 54 | 586 ± 48 | 0 ± 24 | 516 ± 69 | 513 ± 36 | -3 ± 47 | 514 ± 42 | 490 ± 43 | -33 ± 27 | 0.238 | 0.279 | 0.950 |
| Tmax, min | 136 ± 36 | 201 ± 38 | 51 ± 54 | 198 ± 32 | 205 ± 31 | 1 ± 36 | 201 ± 35 | 145 ± 33 | -56 ± 39 | 238 ± 31 | 194 ± 32 | -69 ± 30 | 0.926 | 0.189 | 0.478 |
| AUC, µmol/L x min^3^ | 118 ± 17 | 127 ± 26 | 13 ± 10 | 125 ± 13 | 115 ± 10 | -10 ± 6 | 112 ± 13 | 113 ± 9 | 2 ± 5 | 117 ± 11 | 110 ± 10 | -4 ± 3 | 0.003 | 0.777 | 0.385 |
| iAUC, µmol/L x min^3^ | 98 ± 15 | 109 ± 25 | 14 ± 11 | 105 ± 11 | 95 ± 9 | -10 ± 6 | 93 ± 12 | 86 ± 10 | -7 ± 7 | 96 ± 9 | 92 ± 9 | -3 ± 4 | 0.886 | 0.255 | 0.138 |

^1^Values are untransformed and unadjusted means ± SEMs. For all variables, *n* = 51: Glu298 homozygotes (GG: *n* = 23); Asp298 carriers represents:

Asp298 carrier heterozygotes (GT; *n* = 23) and Asp298 homozygotes (TT; *n* = 5). Time interval for AUC and iAUC: 480 min for all variables, except for

360 min NEFA. AUC, area under the curve; Cmax, maximum concentration; Cmin, minimum concentration; G, genotype; G x T, genotype x treatment;

iAUC, incremental AUC; NEFA, nonesterified fatty acid; T, treatment; TG; triacylglycerol; Tmax, time to reach maximum concentration; Tmin, time to reach minimum concentration; Δ, change-from-pre-intervention.

^2^Linear mixed-model analyses were used to calculate overall treatment effect based on Δ in each 12-wk dietary intervention (calculated by subtracting

week 0 from week 12 values; and week 20 from week 32 values), with adjustments made for fixed effects of baseline values of the assessed outcome measure at the beginning of each dietary period (i.e., the postprandial summary measure for the pre-intervention visit), period, treatment, sex, genotype, age, BMI, and genotype x treatment. Participant was included as a random effect. No period effects were observed in the model for any outcome measure. For all outcome measures, *P* ≤ 0.01 was deemed significant to acknowledge multiplicity.

^3^Indicates data were log transformed prior to analysis.

**Supplemental Figure 1** Flow of participants through the study. % FMD, percentage flow-mediated dilatation response.
